# Differential Shape of Geminivirus Mutant Spectra Across Cultivated and Wild Hosts With Invariant Viral Consensus Sequences

**DOI:** 10.3389/fpls.2018.00932

**Published:** 2018-07-02

**Authors:** Sonia Sánchez-Campos, Guillermo Domínguez-Huerta, Luis Díaz-Martínez, Diego M. Tomás, Jesús Navas-Castillo, Enrique Moriones, Ana Grande-Pérez

**Affiliations:** ^1^Instituto de Hortofruticultura Subtropical y Mediterránea “La Mayora,” Consejo Superior de Investigaciones Científicas-Universidad de Málaga, Estación Experimental “La Mayora,” Algarrobo-Costa, Málaga, Spain; ^2^Instituto de Hortofruticultura Subtropical y Mediterránea “La Mayora,” Consejo Superior de Investigaciones Científicas-Universidad de Málaga, Área de Genética, Facultad de Ciencias, Campus de Teatinos, Málaga, Spain

**Keywords:** geminivirus, begomovirus, tomato yellow leaf curl virus, ssDNA virus quasispecies, viral fitness, begomovirus mutant spectra, host adaptation, emergent viruses

## Abstract

Geminiviruses (family *Geminiviridae*) possess single-stranded circular DNA genomes that are replicated by cellular polymerases in plant host cell nuclei. In their hosts, geminivirus populations behave as ensembles of mutant and recombinant genomes, known as viral quasispecies. This favors the emergence of new geminiviruses with altered host range, facilitating new or more severe diseases or overcoming resistance traits. In warm and temperate areas several whitefly-transmitted geminiviruses of the genus *Begomovirus* cause the tomato yellow leaf curl disease (TYLCD) with significant economic consequences. TYLCD is frequently controlled in commercial tomatoes by using the dominant *Ty-1* resistance gene. Over a 45 day period we have studied the diversification of three begomoviruses causing TYLCD: tomato yellow leaf curl virus (TYLCV), tomato yellow leaf curl Sardinia virus (TYLCSV) and tomato yellow leaf curl Malaga virus (TYLCMaV, a natural recombinant between TYLCV and TYLCSV). Viral quasispecies resulting from inoculation of geminivirus infectious clones were examined in plants of susceptible tomato (*ty-1*/*ty-1*), heterozygous resistant tomato (*Ty-1*/*ty-1*), common bean, and the wild reservoir *Solanum nigrum*. Differences in virus fitness across hosts were observed while viral consensus sequences remained invariant. However, the complexity and heterogeneity of the quasispecies were high, especially in common bean and the wild host. Interestingly, the presence or absence of the *Ty-1* allele in tomato did not lead to differences in begomovirus mutant spectra. However, the fitness decrease of TYLCSV and TYLCV in tomato at 45 dpi might be related to an increase in *CP* (Coat protein) mutation frequency. In *Solanum nigrum* the recombinant TYLCMaV, which showed lower fitness than TYLCSV, at 45 dpi actively explored *Rep* (Replication associated protein) ORF but not the overlapping *C4*. Our results underline the importance of begomovirus mutant spectra during infections. This is especially relevant in the wild reservoir of the viruses, which has the potential to maintain highly diverse mutant spectra without modifying their consensus sequences.

## Introduction

Animal and plant RNA viruses exist as viral quasispecies that adapt to changing environments by evolving rapidly (Domingo et al., 2006, 2012; Roossinck and Schneider, 2006). Viral quasispecies are defined as distributions of nonidentical but related genomes subjected to a continuous process of genetic variation, competition, selection and genetic drift, and which act as a unit of selection (Domingo et al., 2012). Their high genetic diversity in host populations is partially explained by the low replication fidelity of viral RNA-dependent DNA (RdDp) or RNA polymerases (RdRp), which lack proofreading activity (Steinhauer et al., 1992; Domingo and Holland, 1997). Eukaryotic single-stranded DNA (ssDNA) viruses do not encode DNA polymerases (Gutierrez, 2000; Hanley-Bowdoin et al., 2000) and replicate their genomes using unknown host cell polymerases. High mutation frequencies of approximately 10^−4^ mutations per nucleotide as well as substitution rates similar or even higher than those observed for RNA viruses have been found in ssDNA viruses such as animal circoviruses (family *Circoviridae*) (Firth et al., 2009; Harkins et al., 2014; Sarker et al., 2014) and parvoviruses (family *Parvoviridae*) (López-Bueno et al., 2006), or plant geminiviruses (family *Geminiviridae*) (Isnard et al., 1998; Sanz et al., 1999; Ge et al., 2007; Duffy and Holmes, 2008, 2009; Urbino et al., 2008; van der Walt et al., 2008; Harkins et al., 2009) and nanoviruses (family *Nanoviridae*) (Grigoras et al., 2010; Stainton et al., 2015)

Observed genetic diversity within mutant spectra at a given time during viral evolution is the result of natural selection, genetic drift and migration over the continuous generation of viral variants. The heterogeneous composition of the mutant ensemble enables viral emergence and pathogenesis (Coffin, 1996; Domingo et al., 2006, 2012), thus complicating prevention and treatment of viral diseases (Perales et al., 2012). Antiviral or immune-escape mutants present in small proportions of viral populations, for example in human immunodeficiency virus 1 (HIV-1) (Coffin, 1995; Nájera et al., 1995; Henn et al., 2012), hepatitis B virus (HBV) or hepatitis C virus (HCV) (Domingo et al., 2012; Bittar et al., 2013) infections, may be selected and become dominant in quasispecies following antiviral treatment, thus making disease control more difficult (Perales et al., 2014). A widely employed strategy to control plant viruses is the use of host genetic resistance (Boiteux et al., 1993; Monci et al., 2005; Fuchs, 2008; García-Cano et al., 2010). However, given the dynamic nature of viral populations, resistant mutants can emerge and result in epidemics.

Viral emergence requires a sufficient degree of genetic variation in the reservoir host to allow successful infection of the new host after transmission (Parrish et al., 2008; Holmes and Grenfell, 2009; Elena et al., 2011, 2014). Once the virus adapts to the new host, the emergent virus may spread and reach epidemic levels. Thus, knowledge of the genetic structure of host viral populations, and the factors that determine their evolution and host adaptation, are essential for designing robust control strategies for plant viral diseases (Acosta-Leal et al., 2011). The genetic diversity observed in several RNA plant virus quasispecies appears to be host dependent, indicating that virus-host interactions are responsible for the maintenance of variability in mutant spectra (Schneider and Roossinck, 2000, 2001). This diversity might influence viral adaptability to new hosts. It remains to be determined, however, to what extent hosts can play a similar role in shaping quasispecies genetic variability in ssDNA plant viruses.

Begomoviruses (genus *Begomovirus*, family *Geminiviridae*) are emerging pathogens transmitted by the whitefly *Bemisia tabaci* (Gennadius) (*Hemiptera*: *Aleyrodidae*). They are responsible for severe losses of economically important crops such as tomato (*Solanum lycopersicum*), cotton (*Gossypium* spp.) or cassava (*Manihot esculenta*) in tropical, subtropical and temperate regions (Parrish et al., 2008; Rojas and Gilbertson, 2008; Hanssen et al., 2010; Navas-Castillo et al., 2011). The tomato yellow leaf curl disease (TYLCD) is one of the most devastating diseases affecting tomato worldwide (Czosnek and Laterrot, 1997; Díaz-Pendón et al., 2010; Navas-Castillo et al., 2011). TYLCD is caused by a complex of at least 11 different begomoviruses, which produce similar symptoms in infected tomato plants but can differ in host range (Czosnek and Laterrot, 1997; Díaz-Pendón et al., 2010; Navas-Castillo et al., 2011). Most TYLCD-associated viruses have a monopartite genome with coding regions present in both virion (V) and complementary (C) sense strands separated by an intergenic non-coding region (IR). A total of six partially overlapping genes are encoded by monopartite TYLCD-associated virus genomes: *CP* (coat protein, open reading frame or ORF *V1*), *V*2 (movement-like protein, precoat protein), *Rep* (replication associated protein, C1), *TrAP* (transcriptional activator protein, C2), *REn* (replication enhancer protein, C3), and *C4* (involved in systemic movement and a pathogenesis factor) (Czosnek, 2007). TYLCD epidemics have occurred in the Mediterranean Basin since the late 1980s (Czosnek, 2007; Navas-Castillo et al., 2011). In Spain, outbreaks of the disease in the early 1990s were associated with the Spanish (ES) strain of tomato yellow leaf curl Sardinia virus (TYLCSV) (Noris et al., 1994; Navas-Castillo et al., 2011). The subsequent introduction in the late 1990s and early 2000s of the type (also known as Israel, IL) (currently accepted as type member) and Mild (Mld) strains of tomato yellow leaf curl virus (TYLCV) (Noris et al., 1994; Morilla et al., 2004) resulted in new sources of genetic variation, and the emergence of recombinant species such as tomato yellow leaf curl Malaga virus (TYLCMaV), derived from a genetic exchange between isolates of TYLCSV and TYLCV (Navas-Castillo et al., 1997). Although 99% of the TYLCMaV genome sequence is shared with its corresponding parental virus sequences, the virus significantly differs in host range (Morilla et al., 2004; García-Andrés et al., 2007). Thus, TYLCMaV can infect common bean (*Phaseolus vulgaris*) and the wild host *Solanum nigrum*. In contrast, TYLCSV cannot infect common bean (Monci et al., 2002) and the accumulation of TYLCV is strongly impaired in *S. nigrum* (Sánchez-Campos et al., 1999, 2000). None of the latter three viruses induce disease in tomato cultivars carrying the dominant *Ty-1* resistance allele, which has been shown to limit the accumulation of TYLCV, TYLCSV and, to a lesser extent, TYLCMaV (García-Andrés et al., 2006, 2009).

Knowing how hosts affect begomovirus diversity is important to understand the emergence of new begomovirus variants and to assist in designing more durable control strategies. To this end we have monitored the genetic variability of TYLCV, TYLCSV, and TYLCMaV begomovirus mutant spectra for 45 days after the inoculation of susceptible and *Ty-1* resistant tomato, common bean and the wild reservoir *S. nigrum* with single sequence variants of each virus. Our results show that the host can have an important role in driving the fitness and diversity of begomovirus mutant spectra. Differences in absolute fitness of begomovirus in the different hosts existed without any modifications in their consensus sequences. However, quasispecies with high complexity and heterogeneity existed in all four hosts, especially in common bean and the wild host. Interestingly, the presence or absence of the *Ty-1* resistance allele in tomato did not lead to differences in begomovirus mutant spectra. However, in tomato *CP* mutation frequency of TYLCSV and TYLCV increased at 45 dpi, which may be related to their fitness decrease in this host. In the wild host TYLCMaV displayed lower fitness than in the other hosts and actively explored *Rep* ORF but not the overlapping *C4*. Our findings underline the great complexity and heterogeneity of begomovirus mutant spectra and that differences might exist between host plants. This is particularly relevant for the wild reservoir, which hosts a highly diverse begomovirus population containing potentially emergent variants in spite of the stability of their quasispecies consensus sequences.

## Materials and methods

### Plants, virus source, and viral inoculation

**Figure 1:**
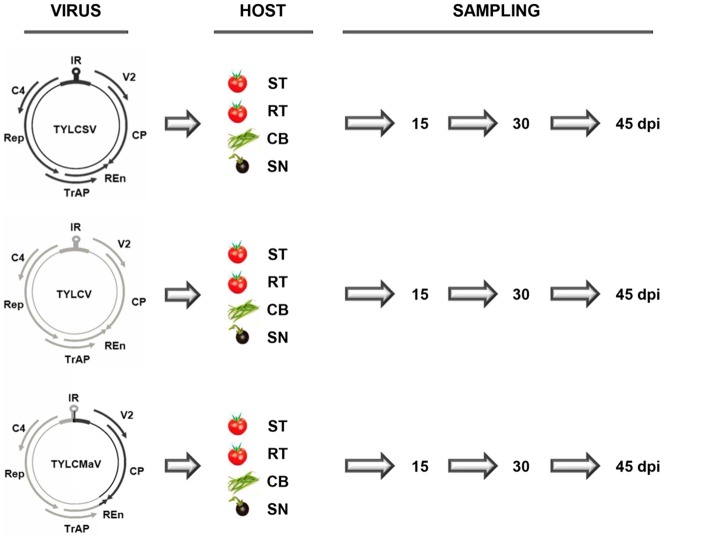
Time-course infections of tomato yellow leaf curl Sardinia virus (TYLCSV), tomato yellow leaf curl virus (TYLCV) and tomato yellow leaf curl Malaga virus (TYLCMaV) infectious clones in susceptible tomato, resistant tomato, common bean and the wild reservoir *Solanum nigrum*. Genomic organization of the viruses showing the two open reading frames (ORFs), *V2*, and *CP*, on the sense strand, and four ORFs, *Rep, TrAP, REn*, and *C4*, on the complementary sense strand. They are separated by an intergenic non-coding region (IR) that contains the origin of replication in a region that adopts a stem-loop structure. Infectious clones and therefore single sequence variants of TYLCSV (black), TYLCV (gray) and recombinant TYLCMaV were used to inoculate three plants each of test hosts: susceptible (ST) and resistant (RT, carrying the *Ty-1* resistance allele in heterozygosis) quasi-isogenic tomato plants, common bean (CB) and the wild reservoir *S. nigrum* (SN). Young apical non-inoculated leaves were collected at the indicated days post inoculation (dpi).

Homozygous TYLCD susceptible (*ty-1*/*ty-1*) and heterozygous TYLCD resistant (*Ty-1*/*ty-1*) tomato quasi-isogenic lines (Monci et al., 2002) (kindly provided by M. J. Díez, Universidad Politécnica de Valencia, Spain), *S. nigrum* (IHSM “La Mayora” germplasm bank) and common bean cv Donna (Nunhems, Haelen, The Netherlands) were used as experimental hosts. Plants at the three-leaf growth stage were inoculated with *Agrobacterium tumefaciens* LBA4404 containing infectious clones, and therefore single sequence variants, of isolates [ES:72:97] of the Mld strain of TYLCV (GenBank accession number AF071228) (García-Andrés et al., 2009), [ES:Mur1:92] of the ES strain of TYLCSV (Z25751) (Navas-Castillo et al., 1999), and [ES:421:99] of TYLCMaV (AF271234) (Noris et al., 1994). These isolates were derived from infected tomato plants except for TYLCMaV, which was derived from an infected common bean plant. TYLCMaV is the result of a genetic exchange between TYLCV-Mld and TYLCSV-ES, with recombinant genome sequences that share 99% nucleotide identity with that of the parental virus sequences. As depicted in Figure 1 in TYLCMaV, the 3′ half of the IR and the virus sense *V2* and the *CP* ORFs derive from TYLCSV, whereas the remainder 5′ half of the IR and the *Rep, C4, TrAP* and most of *REn* ORFs derive from TYLCV, with the 3′ terminal 40 nucleotides of the *REn* ORF derived from TYLCSV (Monci et al., 2002). Viral infectious clones of TYLCSV, TYLCV or TYLCMaV were used to inoculate three plants each of a TYLCD-susceptible tomato, a quasi-isogenic resistant tomato containing the *Ty-1* allele in heterozygosis, common bean and *S. nigrum* (Figure 1). Liquid cultures of *A. tumefaciens* were adjusted to an OD_600_ of 1.00 and 20 μl were inoculated to each plant by the stem-puncture method as described elsewhere (Monci et al., 2002). After agroinoculation, plants were maintained for 45 days in a growth chamber (26°C during the day and 18°C at night, 70% relative humidity, with a 16 h photoperiod at 250 μmol s^−1^m^−2^ photosynthetically active radiation). Systemic virus infection of agroinoculated plants was confirmed on young non-inoculated leaves by tissue-print hybridization using a mixture of digoxigenin (DIG)-labeled probes specific to TYLCV and TYLCSV (Monci et al., 2002) that also recognizes TYLCMaV.

### DNA extraction, virus amplification, cloning, and sequencing

To monitor quasispecies in each infected plant, young apical leaves were harvested at 15, 30, and 45 dpi (Figure 1) and lyophilized. DNA was extracted following the Edwards' procedure (Edwards et al., 1991) with some modifications. From each sample, 8 mg of dried tissue were ground in a 1.5 ml Eppendorf tube with liquid nitrogen, and 400 μl of extraction buffer [200 mM Tris-HCl, 200 mM NaCl, 25 mM EDTA, SDS 0.5% (w/v), pH 7.5, 0.1% β-mercaptoethanol] added. After vortexing at 37°C for 2 min extracts were centrifuged at 12,000 × *g* at room temperature for 5 min. The supernatants were transferred to a new tube and mixed with 1 volume of phenol:chloroform (1:1). The aqueous phase was transferred to a new tube and mixed with 1 volume of isopropanol. After 2 min at room temperature, nucleic acids were recovered by centrifugation at 12,000 × *g* at room temperature for 5 min. The pellet was washed with 70% ethanol, dried and resuspended in 200 μl of RTE buffer (50 mM Tris-HCl pH 8.0, 10 mM EDTA pH 8.0, 0.1% RNase A) by incubation at 65°C for 10 min. Viral DNA from total DNA extracts was amplified by rolling-circle amplification (RCA) with ϕ 29 DNA polymerase according to Inoue-Nagata et al. (Inoue-Nagata et al., 2004) using the TempliPhi DNA Amplification Kit (GE Healthcare). For all samples, 400 to 500 ng/μl of the amplification product measured by UV-spectrophotometry and gel electrophoresis were obtained. Full-lenght genomic consensus sequences were obtained by direct sequencing (Macrogen Inc., South Korea) of RCA products amplified from total DNA extractions. Primers used to sequence consensus sequences of TYLCSV, TYLCV, and TYLCMaV are indicated in Table 1. Sanger sequencing was preferred over NGS due to the difficulty of the latter to discriminate between the error of the technology and the error rate of the viruses, which is paramount for quasispecies analysis.

**Table 1:** Primers used in this study for probe synthesis, sequencing, qPCR and clone analysis.

**Primer**	**Use**	**Template**	**Gene**	**Genomic position**			**Sequence (5′ → 3′)**	**Reference**
				**TYLCSV^a^**	**TYLCV^a^**	**TYLCMaV^a^**		
MA14	Probe synthesis	TYLCSV	*Rep*	2,587–2,604^b^			TGCATTTATTTGAAAACG	Navas-Castillo et al., 1999
MA15	Probe synthesis	TYLCSV	*V2*	162–145			AAAGGATCCCACATATTG	Navas-Castillo et al., 1999
MA20	Sequencing	TYLCV	*CP*		1,019–1,036		TACGCATGCCTCTAATCC	This work
MA30	Probe synthesis	TYLCV	*Rep*		2,564–2,584		GAGCAATTAGGATATGTGAGG	Navas-Castillo et al., 1999
MA31	Probe synthesis	TYLCV	*V2*		168–150		AGTGGGTCCCACATATTGC	Navas-Castillo et al., 1999
MA99	Sequencing	TYLCSV	*CP*	891–908			AAGGAGCAGTGTCTGTTG	Monci et al., 2002
		TYLCMaV	*CP*			891-908		
MA863	Sequencing	TYLCSV	*Rep*	2,217–2,234			CGTAAGCGTCATTGGCTG	
		TYLCV	*Rep*		2,226–2,243			
		TYLCMaV	*Rep*			2,217–2,234		
MA1197	Sequencing	TYLCMalV	*V2*			257–277	TGGGTCATGATCTAATTAGGG	This work
MA1198	Sequencing	TYLCSV	*V2*	257–277			TGGGTCACGATCTAATTAGGG	This work
MA1199	Sequencing	TYLCV	*V2*		263–283		TGGGCCACGATTTAATTAGGG	This work
MA1365	Sequencing	TYLCSV	*Ren*	1,177–1,156			AACAATGTWATYAGAGCAGTTG	This work
		TYLCV	*Ren*		1,186–1,165			
		TYLCMaV	*Ren*			1,177–1,156		
MA1461	Sequencing	TYLCSV	*Rep*	1,703–1,725			CCTGGATTGCAGAGGAAGATAGT	This work
		TYLCV	*Rep*		1,712-1,734			
		TYLCMaV	*Rep*			1,703–1,725		
MA1506	Real-time PCR	TYLCSV	*C4/Rep*	2,225–2,244			TCATTGGCTGTCTGCTGTCC	This work
MA1507	Real-time PCR	TYLCSV	*C4/Rep*	2,462–2,443			ATGGGCAACCTCATCTCCAC	This work
MA1508	Real-time PCR	TYLCV	*C4/Rep*		2,234–2,253		TCATTGGCTGACTGCTGACC	This work
		TYLCMaV	*C4/Rep*			2,225–2,244		
MA1509	Real-time PCR	TYLCV	*C4/Rep*		2,403–2,384		TTCGACCTGGTATCCCCAAG	This work
		TYLCMaV	*C4/Rep*			2,394–2,375		
25S rRNA UNIV (+)	Real-time PCR	Tomato 25S rRNA gene	*-*	-	-	-	ATAACCGCATCAGGTCTCCA	Mason et al., 2008
25S rRNA UNIV(-)	Real-time PCR	Tomato 25S rRNA gene	*-*	-	-	-	CCGAAGTTACGGATCCATTT	Mason et al., 2008
M13 UNIV (+)	Clone PCR analysis	pBSK+	*-*	-	-	-	CGCCAGGGTTTTCCCAGTCACGAC	
M13 UNIV (-)	Clone PCR analysis /sequencing	pBSK+	*-*	-	-	-	TCACACAGGAAACAGCTATGAC	

a *TYLCSV, tomato yellow leaf curl Sardinia virus; TYLCV, tomato yellow leaf curl virus and TYLCMaV, tomato yellow leaf curl Malaga virus*.

b *Nucleotide numbers refer to the sequences of TYLCSV [ES:Mur1:92] (Z25751) (Noris et al., 1994), TYLCV [ES:72:97] (AF071228) (Navas-Castillo et al., 1999), and TYLCMaV [ES:421:99] (AF271234) (Monci et al., 2002)*.

RCA products were digested with single cutter (in viral genome) restriction enzyme *Bam*HI to obtain the linear full-length DNA (c. 2.7 kbp) that was subsequently cloned into pBluescript II SK (+) (Stratagene). The product obtained was transformed into *Escherichia coli* DH5α by electroporation. Transformed colonies were analyzed by PCR using the universal M13 primers (Table 1). Only those clones containing inserts representing the 2.7 kbp full-length genome size were selected for the study. Amplification of molecular clones was also performed using RCA before sequencing. A total of 18–22 clones were sequenced per mutant spectrum from a single plant. Primers MA863 and M13 UNIV (-) were used for all virus isolates (Table 1) to sequence the 5′-end of *Rep* and *C4* ORFs, and the intergenic region (IR) on the C strand. On the virus sense strand the sequenced region comprises the 5′-end of the *V2* ORF and the complete *CP* ORF. Primers MA1197, MA1198, and MA1199 were used for sequencing molecular clones of TYLCMaV, TYLCSV, and, TYLCV, respectively, whereas primer MA1365 was used for all of them.

### Sequence analysis and mutant spectra characterization

EditSeq, SeqBuilder and SeqMan software (DNAStar Inc., USA) were used for sequence assembly and analysis. Mutations were scored relative to the consensus sequence of each mutant spectrum. To analyze virus quasispecies genetic complexity (mutational composition of the ensemble) mutation frequencies were calculated by dividing the number of different mutations (repeated mutations in each quasispecies were computed only once) by the total number of nucleotides sequenced (Domingo et al., 2006). Virus quasispecies genetic complexity was also assessed by analyzing the average genetic distance (*d*), defined as to the average number of mutations per site between any pair of sequences chosen at random from the population. Genetic distances were estimated for each genomic region by Kimura's two-parameter method (Kimura, 1980) in MEGA version 4 (Tamura et al., 2007) in the different host-begomovirus combinations at 15, 30, and 45 dpi. Standard error was calculated by the bootstrap method with 1,000 repeats (Nei and Kumar, 2000).

Mutant spectra heterogeneity was determined using the normalized Shannon entropy (SE) calculation according to the formula − [Σ*i* (*pi x lnpi*)]/*lnN*], where *pi* is the frequency of each sequence in the mutant spectrum and *N* is the total number of sequences compared (Volkenstein, 1994). SE values range from 0 (all sequences are identical) to 1 (all sequences are different).

To analyze the effect of selection on mutant spectra the rate of substitutions per synonymous (*d*_S_) and nonsynonymous (*d*_NS_) site was estimated using the Pamilo-Bianchi-Li method (Pamilo and Bianchi, 1993) implemented in MEGA. The structure-genetic (SG) matrix of Feng et al. (1984) was used to obtain the acceptability values of amino acid changes found in each genomic coding region. The values range from 0 (drastic amino acid changes) to 6 (synonymous replacements) and take into account structural similarities and probabilities of amino acid changes. The probability of occurrence of amino acid replacements found in quasispecies was calculated according to the PAM-250 substitution matrix (Feng and Doolittle, 1996).

Mutations in overlapping genes were only considered for the computation of mutation types or the estimation of *d*, mutation frequency or Shannon entropy of each ORF but not of the entire sequenced zone. Also, since the *Rep* and *C4* ORFs are not in frame, neither *V2* and *CP*, the resulting amino acid changes in the overlapping sequence were considered for the d_NS_/d_S_ calculation.

The statistical significance of differences in ORF mutation frequencies between samples was evaluated by a generalized linear model with logit link function assuming a binary distribution, and followed by post hoc analysis of pairwise comparisons with least square (LSD) correction to test for differences between treatments, using IBM SPSS Statistics v. 22 software (*P* < 0.05 was considered statistically significant). To check for a random distribution of mutations in the overlapping *Rep* and *C4* a Wald–Wolfowitz runs test was conducted.

### Viral fitness determination

To determine absolute fitness the accumulation ability of a virus genotype in a given host, that is, the number of viral genomes produced after a given time (per unit of total DNA extracted from the plant tissue) in apical leaves was measured. Absolute fitness was approximated as the Malthusian growth rate per day, *m*, calculated according to the formula $m=\frac{1}{t}$ *logQ*, where *Q* is the number of pg of begomovirus ssDNA per 100 ng of total plant DNA (Lalić et al., 2011), which is estimated from the qPCR determination of the accumulated viral load in the apical part of each plant being monitored during the 15 days that have elapsed between each collection time (15, 30, and 45 dpi).

Quantification of viral DNA in test plants was carried out on total DNA extracts previously analyzed by agarose gel electrophoresis and UV-spectrophotometry (ND-1,000 spectrophotometer, NanoDrop, Fisher Thermo). Quantification was done in triplicate with a final volume of 20 μl with SYBR Premix Ex Taq Perfect Real Time (Takara), 0.125 μM of specific primers and 10 ng of total DNA. An amplicon of 169 bp within the *Rep* ORF of TYLCV and TYLCMaV was amplified with primers MA1508 and MA1509 (Table 1). In the case of TYLCSV, a fragment of 240 bp also in *Rep* was amplified with primers MA1506 and MA1507. PCR amplification was performed in an iCycler PCR System (BioRad) and consisted of an initial denaturation step at 95°C for 30 s, followed by 40 cycles at 95°C for 5 s and 60°C for 34 s. Specificity of the amplified products was assessed by melting curve analysis. Data from total DNA plant extracts were normalized to the *25S* ribosomal RNA gene amplified using primers 25S rRNA UNIV (+ and –) (Mason et al., 2008; Table 1). To obtain the total number of ssDNA molecules, values were multiplied by two as each standard dsDNA molecule (a phagemid harbors the infectious clone) contains two ssDNAs (virion sense and complementary sense).

For the absolute quantification of TYLCV-like viruses, standard curves were prepared using pBluescript II SK (+) containing full-length viral genomes. Phagemids were serially diluted tenfold in 5 ng/μl of DNA extracted from either non-inoculated tomato, common bean or *S. nigrum* plants, to obtain 10^2^ to 10^9^ viral genome copies per μl. For each PCR system (primer pair and background host DNA), standard curves were obtained by linear regression analysis of the threshold cycle (Cq) values of three standard dilution replicates over the Log of the number of genome copies present in each sample.

A generalized linear model was fitted to the Malthusian growth rate and the log value of viral load data. Post hoc analysis of pairwise comparisons via Bonferroni correction were used to test for differences between hosts at 45 dpi (*P* < 5.56 × 10^−3^) or along the time course of viral infections (*P* < 0.010 or 6.25 × 10^−3^).

## Results

### Host-virus interactions determine differences in viral fitness

**Figure 2:**
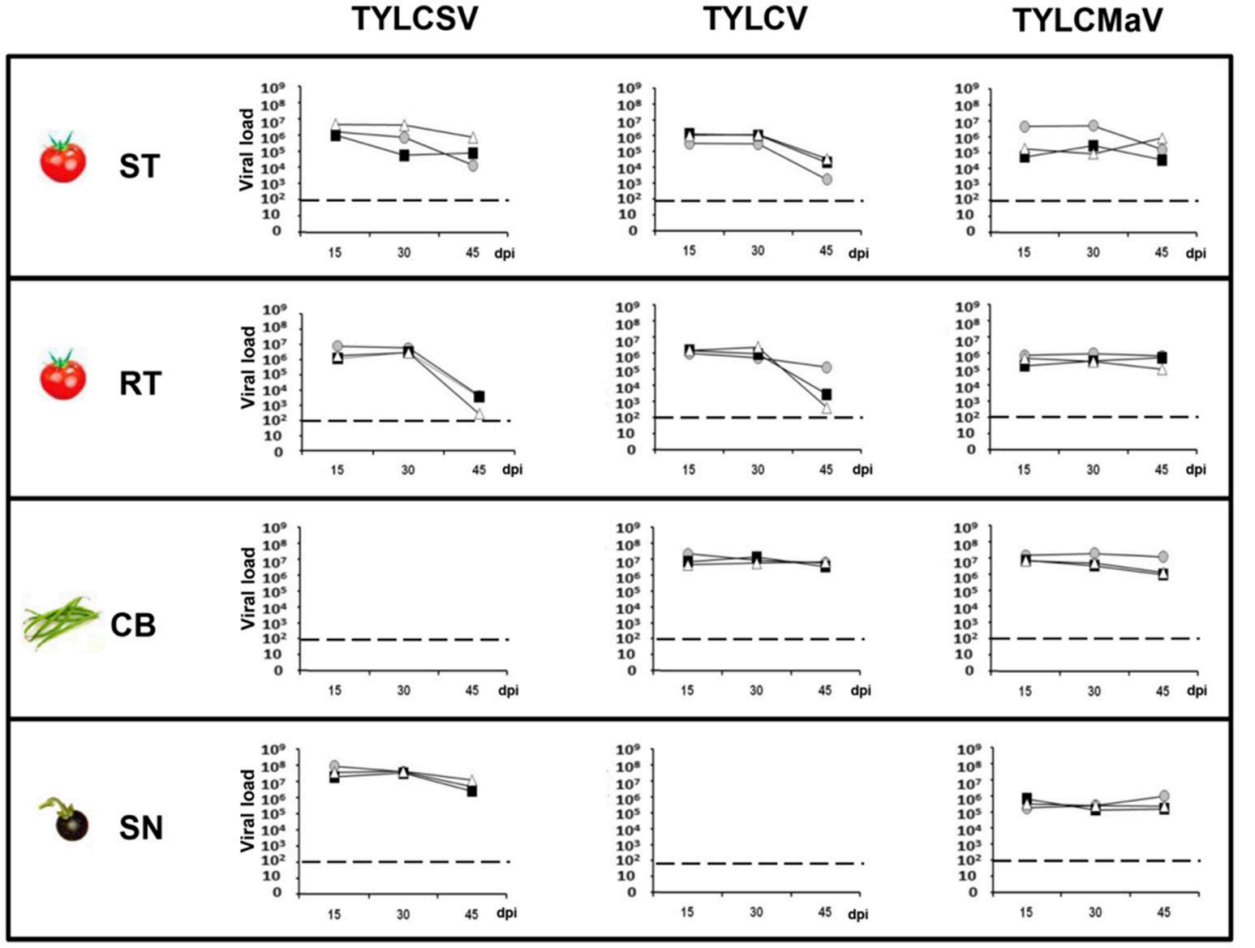
Absolute quantification of begomovirus ssDNA molecules accumulated during time-course analyses of tomato yellow leaf curl Sardinia virus (TYLCSV), tomato yellow leaf curl virus (TYLCV) and tomato yellow leaf curl Malaga virus (TYLCMaV) infections in susceptible tomato (ST), resistant tomato (RT), common bean (CB) and the wild reservoir *Solanum nigrum* (SN). Begomovirus DNA molecules present in the apical leaves of plants infected with infectious begomovirus clones were quantified by qPCR. Where possible, begomovirus ssDNA levels were monitored in three individual systemically infected plants (as judged by hybridization analysis) for each virus/host combination. Apical leaf samples were collected at 15, 30, and 45 days post inoculation (dpi). Graphs show viral loads (number of ssDNA genomes per ng of total DNA) in apical leaf samples from individual test plants, indicating average values from three technical qPCR replicates for each DNA extract. Viral DNA values were normalized to the 25S rRNA gene. No viral accumulation was detected at any time point for TYLCSV/common bean or TYLCV/*S. nigrum* infections either by hybridization or qPCR. Horizontal dotted lines indicate the threshold for qPCR detection. Standard error ranged from 0.01 to 0.001% of the quantified molecules.

To evaluate how the host affects the behavior of the different begomoviruses first we measured the viral load of the three begomoviruses in the four hosts. Under the conditions studied, no effective amplification was possible from young apical non-inoculated leaves for TYLCSV in common bean or for TYLCV in *S. nigrum*. As shown in Figure 2, it was clear that the presence of the *Ty-1* resistance allele repressed accumulation of TYLCSV, and TYLCV to some extent, whereas no such repression was observed for TYLCMaV. Significantly higher TYLCV accumulation was observed in common bean at 45 dpi than in susceptible (510-fold; *P* = 0.005) or resistant (1 × 10^3^-fold; *P* = 0.000) tomato plants. Also, significantly more TYLCSV molecules accumulated in the wild reservoir *S. nigrum* at 45 dpi than in susceptible (60-fold, P = 0.005) or resistant (3.6 × 10^6^-fold, P = 0.0004) tomatoes (Figure 2). Thus, TYLCV and TYLCSV infections in common bean and *S. nigrum*, respectively, resulted in the generation of larger virus populations suggesting higher viral fitness compared to tomato. Our results also suggest better adaptation of TYLCMaV to common bean (with an accumulation level similar to TYLCV) than to *S. nigrum* (much lower accumulation than TYLCSV).

**Figure 3:**
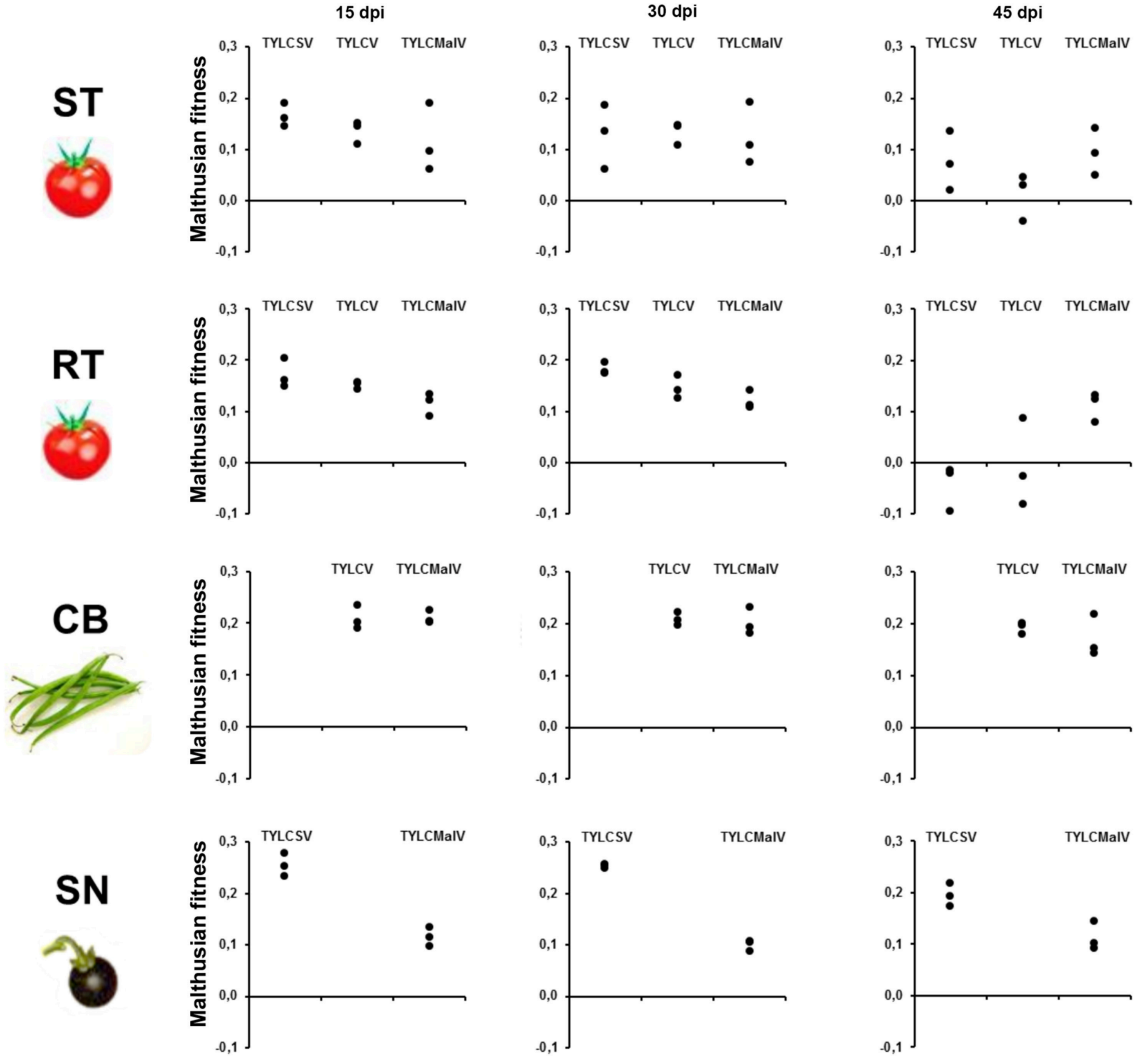
Absolute fitness of begomovirus during time-course analyses of tomato yellow leaf curl Sardinia virus (TYLCSV), tomato yellow leaf curl virus (TYLCV) and tomato yellow leaf curl Malaga virus (TYLCMaV) infections in susceptible tomato (ST), resistant tomato (RT), common bean (CB) and the wild reservoir *Solanum nigrum* (SN). Absolute fitness of begomovirus DNA molecules present in the apical leaves of plants infected with infectious begomovirus clones was approximated by calculating the Malthusian growth rate per day, *m*, calculated according to the formula $m=\frac{1}{t}$ *logQ*, where *Q* is the number of pg of begomovirus ssDNA per 100 ng of total plant DNA. Three individual systemically infected plants (as judged by hybridization analysis) for each virus/host combination were monitored. Apical leaf samples (non-inoculated) were taken at 15, 30 and 45 days post inoculation (dpi). No viral detection was possible at any time point for TYLCSV/common bean or TYLCV/*S. nigrum* infections either by hybridization or qPCR.

To better understand the effect of the different hosts on the three begomoviruses absolute fitness was assessed. Since we used single-sequence variants as inoculum the Malthusian growth rate (m) during viral infection time-courses was calculated as an estimate for absolute fitness (Lalić et al., 2011). The results shown in Figure 3 indicate differences in fitness across hosts during time-course infections. The presence of the *Ty-1* allele significantly restricted the growth rate of TYLCSV (*P* = 0.004) in apical leaves at 45 dpi, and of both TYLCSV and TYLCV during the time-course infection (*P* = 0.000). However, as previously shown (Monci et al., 2002), the presence of the *Ty-1* resistance allele in tomato had no evident effect on TYLCMaV fitness at 45 dpi (*P* > 5.56 × 10^−3^) or during the infection (*P* > 6.25 × 10^−3^). Despite being a recombinant of the former two viruses, TYLCMaV maintained similar fitness throughout the whole experiment in all four hosts (*P* > 6.25 × 10^−3^ or 0.010). Moreover, viral fitness in common bean and in *S. nigrum* was sustained along the experiment (*P* >0.010). In summary, these results suggest better performance of TYLCV and TYLCSV in common bean and *S. nigrum*, respectively, whereas TYLCMaV accumulates similarly well in all hosts, including the resistant tomato.

### High genetic complexity and heterogeneity of TYLCD-associated begomovirus quasispecies

**Figure 4:**
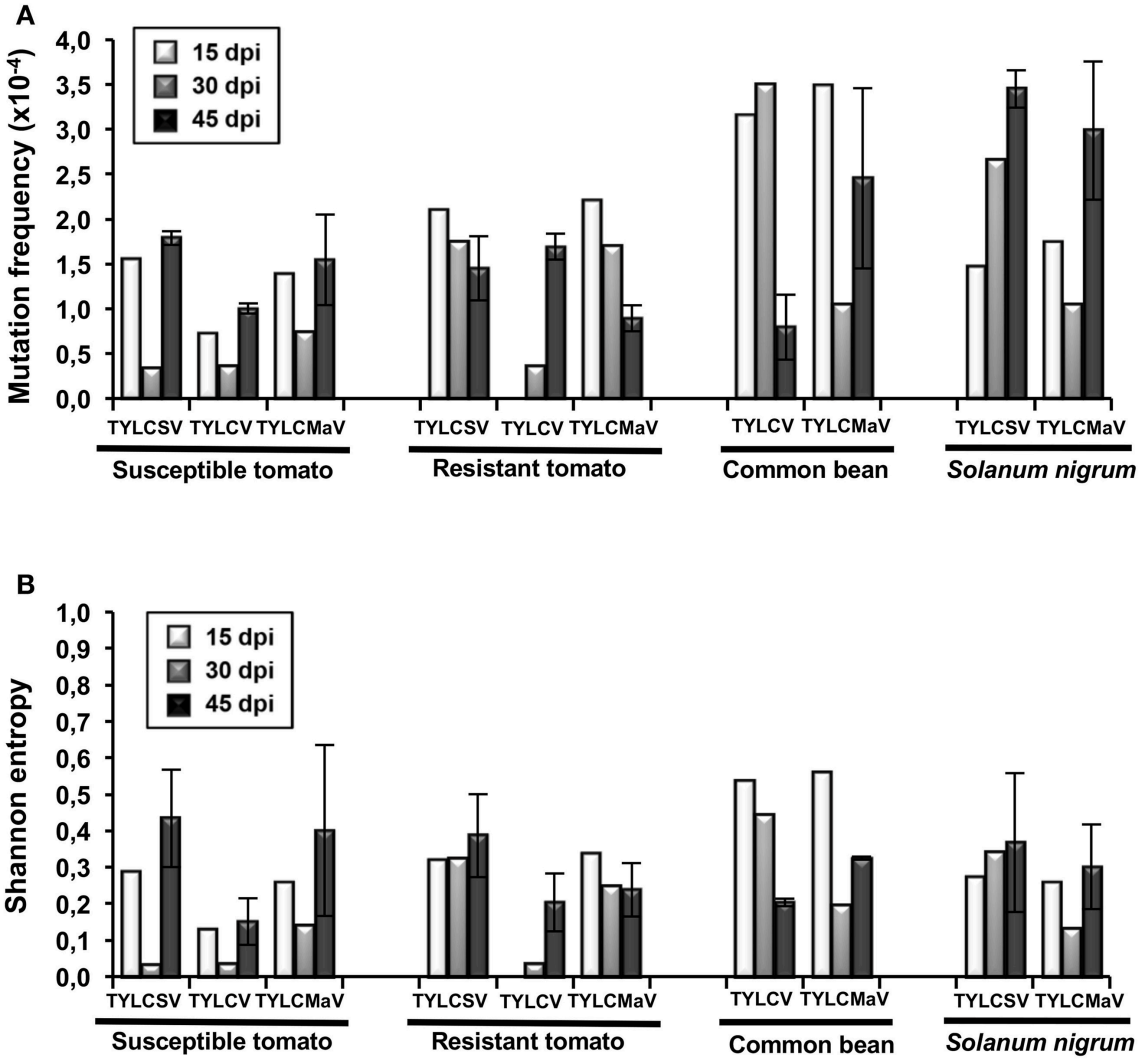
Complexity and heterogeneity of begomovirus quasispecies of tomato yellow leaf curl Sardinia virus (TYLCSV), tomato yellow leaf curl virus (TYLCV) and tomato yellow leaf curl Malaga virus (TYLCMaV) in quasi-isogenic susceptible (*ty-1*/*ty-1*) and resistant (*Ty-1*/*ty-1*) tomato, common bean, and *Solanum nigrum* plants. DNA from young apical leaf samples of the same infected plant per virus-host combination collected at 15, 30, and 45 days post-inoculation (dpi), and of a replicate plant of each virus-host combination at 45 dpi, was amplified by rolling circle amplification (RCA) and the viral DNA products cloned. Sequences of 1,437 nucleotides for TYLCSV, 1,438 nucleotides for TYLCV, and 1,429 nucleotides for TYLCMaV from molecular clones (approximately 20 per quasispecies) were aligned and compared to their consensus sequences. Only mutations (base substitutions and indels) relative to the consensus sequence of each quasispecies were computed. **(A)** Mutation frequencies (mutations per nucleotide) were calculated for each quasispecies. **(B)** Normalized Shannon entropy estimated for each begomovirus quasispecies at the indicated d of experimental evolution. The formula − [Σ*i* (*pi x lnpi*)/*lnN*] was used, where *pi* is the frequency of each sequence in the mutant spectrum and *N* is the total number of sequences compared.

Next we analyzed the quasispecies complexity and heterogeneity of the three begomoviruses during the time-course infections. Specifically, viral quasispecies complexity refers to the composition of the mutant spectrum, which can be described both by mutation frequency and average genetic distance (*d*), whereas viral quasispecies heterogeneity refers to the proportion of different viral genomes in the mutant spectrum, as measured by Shannon entropy (Domingo et al., 2006). DNA samples from one infected plant per virus/host combination at each time point, plus a replicate sample of each combination at 45 dpi, were subjected to rolling circle amplification (RCA) to specifically amplify circular DNA using the high-fidelity φ29 DNA polymerase with a reported error rate of 3–6 × 10^−6^ (Esteban et al., 1993; Nelson et al., 2002). As a measure to reduce amplification bias we amplified 3–7 × 10^8^ input viral molecules. Sequences obtained for each begomovirus region were compared to the consensus sequence to determine mutation frequencies. Despite large variation among viruses, the highest mutation frequency values calculated for the whole sequenced region were detected in common bean and in *S. nigrum* (Figure 4A). When analyzed by genomic region, the mutation frequency of the quasispecies varied from <0.616 × 10^−4^ to 8.91 × 10^−4^ mutations/nt (mut/nt) (Table 2). The most complex region was *V2* (e.g., 8.91 × 10^−4^ for TYLCV in resistant tomato, 8.6 × 10^−4^ and 8.0 × 10^−4^ mut/nt in common bean for TYLCV and TYLCMaV, respectively, or 8.4 × 10^−4^ mut/nt in *S. nigrum* for TYLCSV). In contrast, the least complex mutant spectra were found in the *Rep* and *C4* genomic regions with values generally below 3 × 10^−4^ mut/nt (Table 2). Higher complexity values were found in common bean or *S. nigrum* than in tomato for *CP* or IR in all viral species. Thus, *CP* mutation frequency of TYLCV in common bean was higher than in resistant tomato at 15 dpi (*P* = 0.025), and also higher at 30 dpi than in susceptible (*P* = 0.034) and resistant tomato (*P* = 0.034). Only in TYLCV, *CP* (*P* = 0.030) and *V2* (*P* = 0.045) regions showed more complexity in resistant tomato than in common bean at 45 dpi. Additionally, our results suggest that *CP* and IR were differentially enriched in mutations respect to other regions within a particular quasispecies. In *S. nigrum* at 45 dpi the complexity of *CP* (*P* = 0.001) and IR (*P* = 0.045) of TYLCSV was higher than that of both *Rep* and *C4* regions. Also in *S. nigrum CP* region of TYLCMaV was more complex than *Rep, C4*, and *V2* at 15 (*P* = 0.045) and 30 dpi (*P* = 0.005). In susceptible tomato at 45 dpi the mutation frequency was higher in *CP* than in *C4* (*P* = 0.025) for TYLCSV and in resistant tomato *CP* was more complex than *Rep* (P = 0.001) and *C4* (P = 0.014) for TYLCV.

**Table 2:** Complexity and heterogeneity of tomato yellow leaf curl disease-associated begomovirus populations evolved from single viral sequences in three crop species and a wild host.

**Host^a^**	**Virus^b^**	**Dpi^c^**	***Rep***			***C4***			**IR**			***V2***			***CP***		
			**Mutations /clones**^e^	**Mutation frequency (10–4)**	**Shannon entropy**	**Mutations /clones**	**Mutation frequency (10–4)**	**Shannon entropy**	**Mutations /clones**	**Mutation frequency (10–4)**	**Shannon entropy**	**Mutations /clones**	**Mutation frequency (10–4)**	**Shannon entropy**	**Mutations /clones**	**Mutation frequency (10-4)**	**Shannon entropy**
ST	TYLCSV	15	2/18	3.230	0.147	1/18	2.971	0.037	1/18	1.810	0.037	1/18	2.971	0.037	1/18	0.719	0.037
		30	1/20	1.453	0.034	0/20	<2.674	0	0/20	<1.629	0	0/21	<2.546	0	0/21	<0.616	0
		45(1)	0/19	<1.530	0	0/19	<2.815	0	1/19	1.714	0.047	0/19	<2.815	0	4/19	2.723	0.274
		45(2)^d^	0/16	<1.817	0	0/16	<3.342	0	1/16	2.036	0.084	2/16	6.684	0.167	5/16	4.043	0.405
	TYLCV	15	0/20	<1.453	0	0/20	<2.674	0	1/20	1.572	0.045	0/19	<3.229	0	1/19	0.703	0.035
		30	0/20	<1.453	0	0/20	<2.674	0	0/20	<1.572	0	1/20	3.067	0.034	1/20	0.668	0.034
		45(1)	0/19	<1.530	0	0/19	<2.815	0	0/19	<1.655	0	0/21	<2.506	0	3/21	1.841	0.186
		45(2)	0/20	<1.451	0	0/20	<2.673	0	0/20	<1.628	0	0/20	<2.631	0	0/20	<6.440	0
	TYLCMaV	15	1/20	1.453	0.034	0/20	<2.674	0	1/20	1.603	0.034	0/20	<2.674	0	2/20	1.294	0.132
		30	0/20	<1.453	0	0/20	<2.674	0	2/20	3.205	0.132	0/18	<2.971	0	0/18	<0.719	0
		45(1)	0/22	<1.321	0	0/22	<2.431	0	2/22	2.914	0.119	0/22	<2.431	0	4/22	2.352	0.235
		45(2)	2/18	1.615	0.147	1/18	2.971	0.074	1/18	1.810	0.074	0/18	<2.971	0	0/18	<0.719	0
RT	TYLCSV	15	2/20	2.907	0.034	2/20	5.348	0.034	3/20	4.886	0.196	0/20	<2.674	0	1/20	0.647	0.034
		30	2/20	2.907	0.132	1/20	2.674	0.034	1/20	1.629	0.034	1/20	2.674	0.034	2/20	1.294	0.132
		45(1)	1/20	1.453	0.034	0/20	<2.674	0	0/20	<1.629	0	2/21	5.093	0.125	4/21	2.464	0.247
		45(2)	1/17	1.710	0.078	0/17	<3.146	0	1/17	1.916	0.078	0/17	<3.146	0	1/17	0.761	0.078
	TYLCV	15	0/20	<1.453	0	0/20	<2.674	0	0/20	<1.572	0	0/20	<3.067	0	0/20	<0.657	0
		30	0/20	<1.453	0	0/20	<2.674	0	0/20	<1.572	0	1/20	2.857	0.034	1/20	0.657	0.034
		45(1)	0/20	<1.453	0	0/20	<2.674	0	0/20	<1.572	0	1/19	3.229	0.035	5/19	3.391	0.274
		45(2)	1/18	1.615	0.074	1/18	2.971	0.074	0/18	<1.810	0	3/18	8.913	0.219	7/18	5.031	0.493
	TYLCMaV	15	2/19	3.060	0.139	1/19	2.815	0.035	3/19	5.061	0.139	0/19	<2.815	0	1/19	0.681	0.035
		30	3/20	4.360	0.196	1/20	2.674	0.034	1/20	1.603	0.034	0/21	<2.546	0	1/21	0.616	0.032
		45(1)	0/21	<1.384	0	0/21	<2.546	0	1/21	1.526	0.032	1/21	2.546	0.032	2/21	1.232	0.125
		45(2)	2/18	3.230	0.147	1/18	2.971	0.074	0/18	<1.810	0	0/18	<1.810	0	2/18	1.437	0.147
CB	TYLCV	15	2/20	2.907	0.132	1/20	2.674	0.034	2/20	3.145	0.244	1/20	2.857	0.034	5/20	3.285	0.299
		30	2/20	2.907	0.132	1/20	2.674	0.034	2/20	3.145	0.132	3/20	8.571	0.196	7/20	4.599	0.322
		45(1)	1/20	1.453	0.034	0/20	<2.674	0	0/20	<1.572	0	0/20	<2.857	0	2/20	1.314	0.132
		45(2)	0/13	<2.906	0	0/13	<4.114	0	0/13	<2.419	0	0/13	<4.396	0	1/13	1.011	0.105
	TYLCMaV	15	3/20	4.360	0.034	0/20	<2.674	0	2/20	3.205	0.132	3/20	8.021	0.132	5/20	3.234	0.260
		30	1/20	1.453	0.034	1/20	2.674	0.034	0/20	<1.603	0	0/20	<2.674	0	2/20	1.294	0.132
		45(1)	1/20	1.453	0.196	1/20	2.674	0.034	0/20	<1.603	0	1/20	2.674	0.034	4/20	2.587	0.260
		45(2)	4/19	6.120	0.273	1/19	2.815	0.070	3/19	4.965	0.207	0/19	<3.008	0	5/19	3.458	0.339
SN	TYLCSV	15	0/19	<1.530	0	0/19	<2.815	0	1/19	1.714	0.035	1/19	2.815	0.035	3/19	2.043	0.207
		30	1/19	1.530	0.035	1/19	2.815	0.035	3/19	5.143	0.035	1/18	2.971	0.037	3/18	2.156	0.219
		45(1)	0/20	<1.453	0	0/20	<2.674	0	3/20	4.886	0.196	3/19	8.444	0.207	7/19	4.766	0.340
		45(2)	0/19	<1.530	0	0/19	<2.815	0	1/19	1.714	0.070	2/19	5.629	0.139	5/19	3.404	0.139
	TYLCMaV	15	0/20	<1.453	0	0/20	<2.674	0	1/20	1.603	0.034	0/20	<2.674	0	4/20	2.587	0.260
		30	0/20	<1.453	0	0/20	<2.674	0	1/20	1.603	0.034	0/20	<2.674	0	2/20	1.294	0.132
		45(1)	2/20	2.907	0.132	1/20	2.674	0.034	3/20	4.808	0.196	0/20	<2.674	0	2/20	1.294	0.132
		45(2)	2/8	7.267	0.353	1/8	6.684	0.181	1/8	4.072	0.181	0/8	<6.684	0	0/8	<1.617	0

a *ST, susceptible tomato; RT, resistant tomato; CB, common bean; SN, Solanum nigrum*.

b *Days post inoculation*.

c *TYLCSV, tomato yellow leaf curl Sardinia virus; TYLCV, tomato yellow leaf curl virus; and TYLCMaV, tomato yellow leaf curl Malaga virus*.

d *Numbers in brackets indicate replicate sample*.

e Total number of mutations found in the indicated number of clones analyzed in the quasispecies

Quasispecies genetic complexity was also assessed by analyzing average genetic distance *(d*) values. As shown in Table 3, *d* values varied from 0.00012 to 0.00173. The maximum genetic distance values for the four coding regions and the IR were found in the *V2* region (especially for common bean and *S. nigrum*). High *d* values were also observed for *C4* in some cases (e.g., in resistant tomato for TYLCSV at 15 dpi or in common bean for TYLCV at 30 dpi).

**Table 3:** Average pairwise genetic distances (*d*) estimated by Kimura's two-parameter method for begomovirus *Rep, C4, V2*, and *CP* coding regions, and the noncoding IR, after 45 days of tomato yellow leaf curl Sardinia virus (TYLCSV), tomato yellow leaf curl virus (TYLCV) and tomato yellow leaf curl Malaga virus (TYLCMaV) infection in susceptible (ST) and resistant tomato (RT), common bean (*Phaseolus vulgaris*, CB) and *Solanum nigrum* (SN) hosts.

			**Genomic Region**									
**Host**	**Virus**	**Dpi**	***Rep***		***C4***		**IR**		***V2***		***CP***	
			***d***	SE^*^	***d***	**SE**	***d***	**SE**	***d***	**SE**	***d***	**SE**
ST	TYLCSV	15	0.00065	0.00047	0.00059	0.00061	0.00036	0.00035	0.00060	0.00058	0.00014	0.00014
		30	0.00000	0.00000	0.00000	0.00000	0.00000	0.00000	0.00000	0.00000	0.00000	0.00000
		45 (1)	0.00000	0.00000	0.00000	0.00000	0.00000	0.00000	0.00000	0.00000	0.00068	0.00028
		45 (2)	0.00035	0.00035	0.00064	0.00062	0.00082	0.00057	0.00000	0.00000	0.00032	0.00032
	TYLCV	15	0.00000	0.00000	0.00000	0.00000	0.00060	0.00057	0.00000	0.00000	0.00014	0.00014
		30	0.00000	0.00000	0.00000	0.00000	0.00000	0.00000	0.00062	0.00060	0.00013	0.00013
		45 (1)	0.00000	0.00000	0.00000	0.00000	0.00000	0.00000	0.00000	0.00000	0.00037	0.00021
		45 (2)	0.00000	0.00000	0.00000	0.00000	0.00000	0.00000	0.00000	0.00000	0.00037	0.00038
	TYLCMaV	15	0.00055	0.00054	0.00000	0.00000	0.00032	0.00031	0.00000	0.00000	0.00026	0.00017
		30	0.00000	0.00000	0.00000	0.00000	0.00065	0.00045	0.00000	0.00000	0.00000	0.00000
		45 (1)	0.00000	0.00000	0.00000	0.00000	0.00029	0.00029	0.00000	0.00000	0.00035	0.00019
		45 (2)	0.00031	0.00032	0.00056	0.00054	0.00071	0.00048	0.00000	0.00000	0.00000	0.00000
RT	TYLCSV	15	0.00058	0.00039	0.00107	0.00073	0.00066	0.00047	0.00000	0.00000	0.00013	0.00012
		30	0.00058	0.00041	0.00053	0.00052	0.00033	0.00031	0.00054	0.00053	0.00026	0.00018
		45 (1)	0.00029	0.00027	0.00000	0.00000	0.00000	0.00000	0.00103	0.00071	0.00049	0.00023
		45 (2)	0.00038	0.00036	0.00000	0.00000	0.00041	0.00040	0.00000	0.00000	0.00014	0.00014
	TYLCV	15	0.00000	0.00000	0.00000	0.00000	0.00000	0.00000	0.00000	0.00000	0.00000	0.00000
		30	0.00000	0.00000	0.00000	0.00000	0.00000	0.00000	0.00058	0.00056	0.00013	0.00012
		45 (1)	0.00000	0.00000	0.00000	0.00000	0.00000	0.00000	0.00056	0.00054	0.00054	0.00026
		45 (2)	0.00036	0.00035	0.00075	0.00075	0.00036	0.00034	0.00121	0.00081	0.00155	0.00055
	TYLCMaV	15	0.00061	0.00041	0.00056	0.00054	0.00068	0.00048	0.00000	0.00000	0.00014	0.00013
		30	0.00058	0.00040	0.00053	0.00052	0.00032	0.00032	0.00000	0.00000	0.00012	0.00012
		45 (1)	0.00000	0.00000	0.00000	0.00000	0.00031	0.00030	0.00049	0.00048	0.00024	0.00016
		45 (2)	0.00063	0.00042	0.00056	0.00056	0.00000	0.00000	0.00000	0.00000	0.00015	0.00015
CB	TYLCV	15	0.00058	0.00039	0.00053	0.00053	0.00145	0.00099	0.00109	0.00108	0.00089	0.00043
		30	0.00058	0.00039	0.00107	0.00076	0.00032	0.00030	0.00173	0.00099	0.00092	0.00036
		45 (1)	0.00029	0.00028	0.00000	0.00000	0.00000	0.00000	0.00000	0.00000	0.00026	0.00019
		45 (2)	0.00000	0.00000	0.00000	0.00000	0.00000	0.00000	0.00000	0.00000	0.00020	0.00020
	TYLCMaV	15	0.00087	0.00050	0.00000	0.00000	0.00064	0.00043	0.00162	0.00093	0.00065	0.00027
		30	0.00029	0.00028	0.00053	0.00053	0.00000	0.00000	0.00000	0.00000	0.00039	0.00022
		45 (1)	0.00029	0.00028	0.00053	0.00052	0.00000	0.00000	0.00054	0.00053	0.00052	0.00025
		45 (2)	0.00256	0.00152	0.00056	0.00056	0.00000	0.00000	0.00000	0.00000	0.00015	0.00015
SN	TYLCSV	15	0.00000	0.00000	0.00000	0.00000	0.00035	0.00034	0.00057	0.00056	0.00041	0.00023
		30	0.00031	0.00030	0.00056	0.00054	0.00104	0.00059	0.00060	0.00059	0.00043	0.00022
		45 (1)	0.00000	0.00000	0.00000	0.00000	0.00098	0.00054	0.00171	0.00094	0.00095	0.00035
		45 (2)	0.00000	0.00000	0.00000	0.00000	0.00073	0.00050	0.00140	0.00081	0.00066	0.00036
	TYLCMaV	15	0.00000	0.00000	0.00000	0.00000	0.00032	0.00031	0.00000	0.00000	0.00052	0.00025
		30	0.00000	0.00000	0.00000	0.00000	0.00032	0.00032	0.00000	0.00000	0.00026	0.00017
		45 (1)	0.00040	0.00040	0.00000	0.00000	0.00092	0.00053	0.00000	0.00000	0.00000	0.00000
		45 (2)	0.00082	0.00081	0.00000	0.00000	0.00071	0.00068	0.00000	0.00000	0.00000	0.00000

* *SE, standard error*.

Begomovirus mutant spectra heterogeneity was evaluated by calculating the normalized Shannon entropy of viral quasispecies. Shannon entropy values calculated for the whole sequenced region were highest in common bean at 15 dpi and no difference between hosts were detected a later times post infection (Figure 4B). Susceptible tomato begomovirus quasispecies were the least heterogeneous, followed by those of resistant tomato and *S. nigrum*. Analyzed by genomic region, as shown in Table 2, Shannon entropy values ranged from 0 to 0.340. The *CP* region showed the highest heterogeneity in all four hosts and viruses, although this was especially pronounced in common bean and *S. nigrum*. Conversely, the lowest Shannon entropy values in the four hosts were detected in the *C4* genomic region, except for TYLCSV at 15 dpi in resistant tomato. Therefore, heterogeneity of quasispecies was high in all host/begomovirus combinations, with the most heterogeneous populations exhibited by common bean and *S. nigrum*. Notably, the wild host *S. nigrum* contained the most heterogeneous populations at later infection time points for the viruses that were able to infect it. In general, mutation frequency, *d* and Shannon entropy values at 45 dpi were similar for replicates 1 and 2 for each virus/host combination.

Altogether, our results support the idea that begomovirus infections are characterized by the generation of highly complex and heterogeneous ssDNA mutant spectra in the host species tested, especially in common bean and the wild reservoir *S. nigrum. V2* region supported the most complex spectra, *CP* was the most heterogeneous, while *C4* exhibited both the least complexity and least heterogeneity. Interestingly, despite the high genetic complexity and heterogeneity of TYLCD-associated begomovirus quasispecies, no changes were detected in their whole genomic consensus sequences in any of the four hosts at any time point during infection.

### Analysis of mutations suggests that the *CP* region is the most susceptible to change

Mutation data, comprising 128 base substitutions and 16 indels (complete list shown in Table 4), were pooled together irrespective of sampling time, virus or host. Mutation types and their frequency (%) relative to the number of changes found in a given genomic region or the whole sequenced zone are indicated in Table 5. Independent analysis of *Rep, C4*, IR, *V2*, and *CP* regions, shows that the most abundant mutations were base substitutions (86.7–100%), with indels only absent in *C4*. The IR contained the highest fraction of insertions, representing 11.1% of all mutations in the region. On the other hand, the highest fraction of deletions was detected in *Rep* (5.3% of total mutations). Of the base substitutions, the most common transition was C → T, which was highly enriched in *C4* and *V2* regions. G → A was the next most common transition, although it was less common in the IR and absent from *C4*. With regards to transversions, G → T was the most abundant. Conversely, A → T and A → C and G → T transversions were exceptionally frequent in the IR compared to the other four regions. Interestingly, the transition:transversion ratio was close to 1 in all regions. Transition:transversion ratios in DNA-based organisms (Begun et al., 2007; Hodgkinson and Eyre-Walker, 2010) as well as in RNA and dsDNA viruses (Duchêne et al., 2015) are over 2. While it has not been shown for ssDNA viruses, lower ratios might suggest that in all the begomovirus regions analyzed, especially in the IR and *CP* (with ratios of 0.9), either negative selection is removing transitions or transversions are favored. Nonsynonymous changes were more frequent than synonymous mutations in all regions, ranging from 65.4 to 79.2% of total mutations in *Rep* and *V2*, respectively.

**Table 4:** Nucleotide and amino acid changes found in genomic regions of begomovirus mutant clones.

**Host^b^**	**dpi^c^**	**TYLCSV**^**a**^							**TYLCV**^**a**^							**TYLCMaV**^**a**^						
		**Coding region** ***Rep-C4***			**Coding region** ***V2-CP***			**IR**	**Coding region** ***Rep-C4***			**Coding region** ***V2-CP***			**IR**	**Coding region** ***Rep-C4***			**Coding region** ***V2-CP***			**IR**
		**Nt**	***Rep***	***C4***	**Nt**	***V2***	***CP***	**Nt**	**Nt**	***Rep***	***C4***	**Nt**	***V2***	***CP***	**Nt**	**Nt**	***Rep***	***C4***	**Nt**	***V2***	***CP***	**Nt**
ST	15	C2344T	Q92Q	R40K	C465G	A91G	P53A	A2636C				C585T	-	R82C	C23T (2)	G2604A	R6C (2)	-	G545T	-	Q79H	A140T
		C2611G	Q3H	-															G901A	-	C198Y	
	30							A2691Del				G478T	Q107H	R46I								T2716C
																						C25T
	45(1)^d^				G533A	-	Q75Q	Ins141A (2)				C540G	-	Q76E					C500T	-	V64V	A2741del
					G848T	-	M180I					C874T	-	T187I					G662T	-	G118G	T2750C
					T882A	-	S192T					A961T	-	Q216L					G670del	-	Frameshift	
					T1022C	-	S238S												G678A	-	E124K	
	45(2)				G716A	-	M136I					T546C	-	Y78H		C2411T	C70Y	A18T				
					C1006T	-	A233V															C2751T
		G2380C	S80S	P28R																		G2772T
								G2622T														
								G2728A														
RT	15	T2300C	D107G	T55A	A527G	-	K73K	G2756A								T2431A	Q63H	N11I	G830T	-	R174M	A2764T
		G2314C	S102S	P50R				G98C								G2467A	H51H	-				G2780A
								Ins141A														Ins141A
	30	G2428T	F64L	S12Stop	C407T	P72S	A33A	A2750T				C409T	C85C	A27V		C2289T	V111I	P58P	G761T	-	M151I	A2741C
		G2535T	L29I	-	C1036T	-	A243V									C2466T	E52K	-				
																Ins2519T	Frameshift	-				
																						
	45(1)	G2551T	L23L	-	C409A	P72P	P34H					G499C	K115N	S62T					G482A	V97I	M58I	A64G
					A489C	K99T	S61R					Ins661T	-	Frameshift					T512G	-	C68W	
					G711A	-	V135I					G854A	-	V180V								
					C879A	-	P191T					T917G	-	V201V								
												A1085C	-	S257S								
	45(2)				T788A	-	F160L					C341T	N62N	I9I					C500del	-	Frameshift	
		G2467T	H51Q	-								ins417C	Frameshift	Frameshift					G836A	-	R176R	
								G2664T				A484C	I110I	Y57S		A2435T	I62N	S10T				
												G701A	-	K129K		T2607A	K5Stop	-				
												C797G	-	F161L								
												G980T	-	E222D								
												C1064T	-	I250I								
									C2395T	L78L	W26Stop											
CB	15								G2342T	S96Y	P44T	G504T	Stop117L (2)	D59Y (2)	G2698T (3)	G2517T	L35I	-	G356T	G55C	K16N	C2724T
									C2486T	R48K	-	C655T	-	S109L	A79G (2)	T2524G	L32L	-	C360T	S56L	R18C	A2756C
												C731T	-	F134F		G2611A	P3P	-	T424C	H77H	I39T	
												C820T	-	T164I					A612T	-	T102S	
												A930T	-	R201Stop (2)					C1018T	-	A237V	
	30								C2376G	A85P	Q32H	C346A	F64L	S6Y	A2757T	C2277T	E115K	V62V	C751A	-	T148N	
												C395T	Q81Stop	Y22Y	Ins2775A				G998A	-	R202M	
												C455T	Q101Stop	Y42Y								
												A568C	-	K80T								
												G597T	-	D90L								
												A1062C	-	I245L								
												T1069A	-	F247Y								
	45(1)								G2479T	L50L	-	T1025C	-	H232H		C2317T	K101K	S49N	G382A	Q63Q	S25N	
												T1029A	-	S234T					G621A	-	V105I	
																			G739T	-	R144M	
																			C839T	-	Y177Y	
	45(2)											T799C	-	D162A					G547T	-	R80L	
																			A553T	-	D82V	
																			G624T	-	G106C	
																			A961T	-	A218V	
																			G1013T	-	T235T	
																A2323G	D99D	M47T				
																T2476A	R48S	-				
																C2527T	Q31Q	-				
																C2617T	M1I	-				
																						C2663T
																						T2689G
																						C2737T
SN	15				T458A	S89T	Y50Stop	C22T											A648T	-	I114F	G41A
					G716A	-	M136I												C650A	-	I114I	
					C1075T	-	T256I												T720G	-	F138V	
																			C805T	-	T166M	
	30	A2328G	S98P	A45A	C360T	S56L	R18C	A100T											T518A	-	G70G	A54C
					G822T	-	D172Y	A104T											C650A	-	I114I	
					G913T	-	R202M	Ins106T														
	45(1)				C313A	A40A	P2Q	T2732A								Del 2307-2414	Frameshift	Frameshift	Del 537-736	-	Frameshift	C2758T
					G405A	C71Y	A33T	A2739T								G2467A	H51H	-	Del 541-747	-	Frameshift	T69C
					C416T	P75S	V36V	C105T														T82G
					A654G	-	I116V															
					C879T	-	P191S															
					T947C	-	N213N															
					C948A	-	H214N															
	45(2)				C406T	C86C	A33V									A2288T	V111D	S59T				
					C408T	P87L	P34S									G2570A	T17K	-				
					G730T	-	R141L															C2844G
					G867T	-	V187L															
					C1069A	-	A254M															
								C2842A														
																						

a *TYLCSV, tomato yellow leaf curl Sardinia virus; TYLCV, tomato yellow leaf curl virus and TYLCMaV, tomato yellow leaf curl Malaga virus. Nucleotide numbers based on the sequences of TYLCSV (GenBank Z25751), TYLCV (GenBank AF071228) and TYLCMaV (GenBank AF271234)*.

b *ST, susceptible tomato; RT, resistant tomato; CB, common bean; SN, Solanum nigrum*.

c *Days post inoculation*.

d *Numbers in brackets indicate replicate sample*.

**Table 5:** Number and relative frequency (%) of the different types of mutations found in begomovirus mutant spectra.

**Type of mutation^a^**	***Rep***^**b**^		***C4***		**IR**		***V2***		***CP***		**TOTAL**	
	**n**	**%**	**n**	**%**	**n**	**%**	**n**	**%**	**n**	**%**	**n**	**%**
**TRANSITIONS**												
A → G	2	7.7	2	18.2	2	5.7	0	0	2	2.6	5	3.6
G → A	5	19.2	0	0.0	4	11.4	4	19.0	13	16.7	18	12.9
C → T	10	38.5	6	54.5	9	25.7	10	47.6	23	29.5	30	21.6
T → C	1	3.8	1	9.1	3	8.6	1	4.8	6	7.7	8	5.8
Total	18	69.2	9	81.8	18	51.4	15	71.4	44	56.4	61	43.9
**TRANSVERSIONS**												
A → C	0	0.0	0	0.0	4	11.4	2	9.5	5	6.4	8	5.8
C → A	0	0.0	0	0.0	1	2.9	3	14.3	9	11.5	8	5.8
A → T	0	0.0	2	18.2	7	20.0	0	0.0	6	7.7	11	7.9
T → A	3	11.5	1	9.1	1	2.9	2	9.5	5	6.4	7	5.0
C → G	4	15.4	1	9.1	1	2.9	1	4.8	3	3.8	4	2.9
G → C	1	3.8	2	18.2	1	2.9	1	4.8	1	1.3	3	2.2
G → T	7	26.9	2	18.2	4	11.4	3	14.3	18	23.1	19	13.7
T → G	2	7.7	0	0.0	2	5.7	0	0.0	3	3.8	7	5.0
Total	17	65.4	8	72.7	21	60.0	12	57.1	39	50.0	67	48.2
Ts/Tv^c^	1.1		1.1		0.9		1.3		1.1		0.9	
Substitutions	35	92.1	17	100.0	39	86.7	27	93.1	83	94.3	128	88.9
Insertions	1	2.6	0	0.0	5	11.1	1	3.4	2	2.3	9	6.3
Deletions	2	5.3	0	0.0	1	2.2	1	3.4	3	3.4	7	4.9
Total	38		17		45		29		88		144	
Nonsynonymous	17	65.4	10	76.9	0	0.0	19	79.2	60	75.0	106	74.1
Missense	14	53.8	8	61.5	Na^d^	Na	16	66.7	54	67.5	92	64.3
Nonsense	0	0.0	1	7.7	Na	Na	2	8.3	2	2.5	5	3.5
Frameshift	3	11.5	1	7.7	Na	Na	0	0.0	4	5.0	8	5.6
Readthrough	0	0.0	0	0.0	Na	Na	1	4.2	0	0.0	1	0.7
Synonymous	9	34.6	3	23.1	Na	Na	5	20.8	20	25.0	37	25.9

a *Mutations found in tomato yellow leaf curl Sardinia virus, tomato yellow leaf curl virus and tomato yellow leaf curl Malaga virus mutant spectra after 15, 30, and 45 days post inoculation of infectious clones. Mutations were scored with respect to the consensus sequence*.

b *Genomic regions were defined by the following nucleotide positions: Rep from 2,262 to 2,619, C4 from 2,262 to 2,462, IR from 2,619 to 149, V2 from 309 to 496 and CP from 309 to 1,082. Positions refer to the TYLCSV sequence [ES:Mur1:92] (Z25751) (Noris et al., 1994)*.

c *Ts:Tv, transition:transversion ratio*.

d *Na, not applicable*.

**Figure 5:**
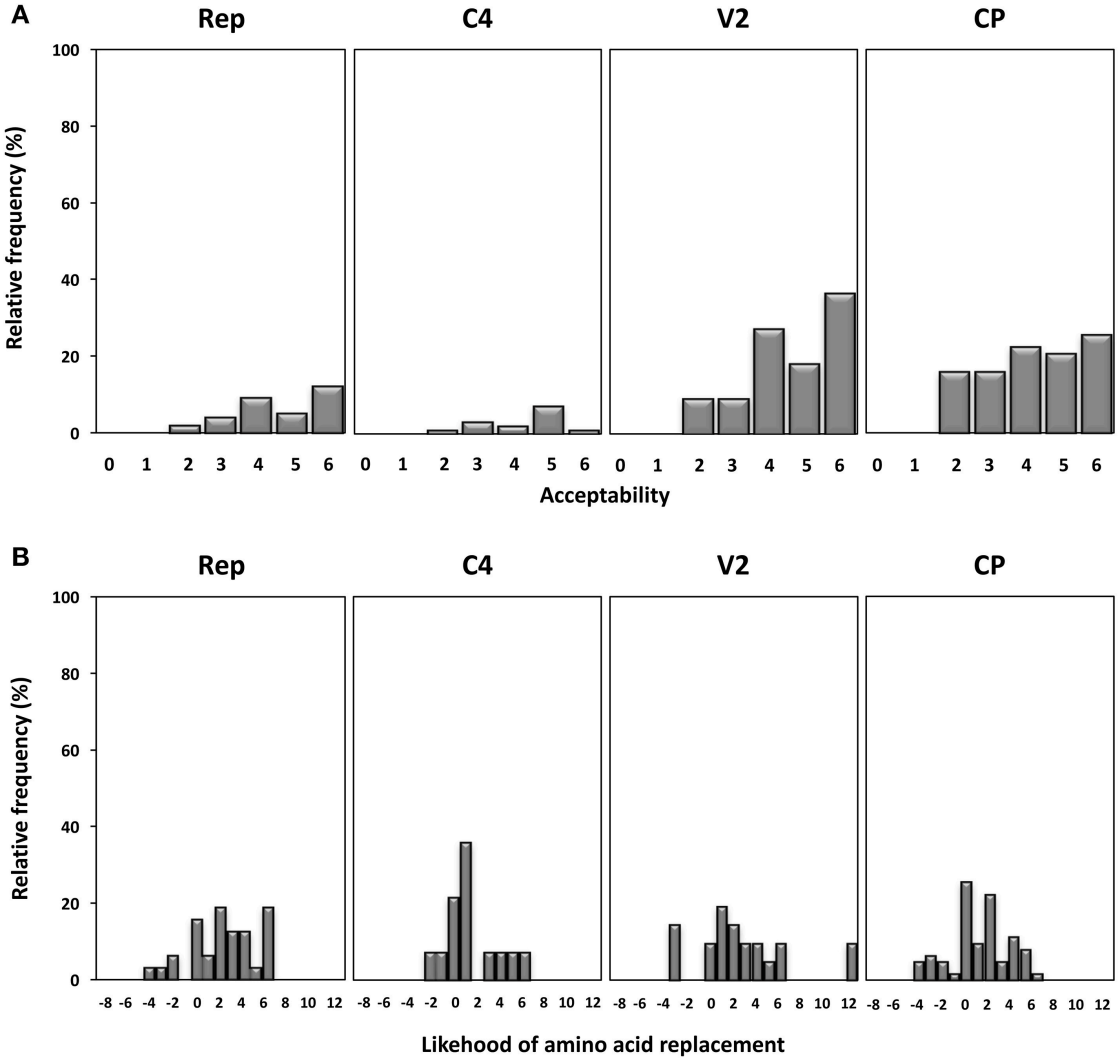
Acceptability and occurrence probability of the amino acid replacements observed in begomovirus quasispecies of tomato yellow leaf curl Sardinia virus (TYLCSV), tomato yellow leaf curl virus (TYLCV) and tomato yellow leaf curl Malaga virus (TYLCMaV) in quasi-isogenic susceptible (*ty-1*/*ty-1*) and resistant (*Ty-1*/*ty-1*) tomato, common bean, and *Solanum nigrum* plants. **(A)** Acceptability values of amino acid changes found in each genomic coding region determined according to the structure-genetic (SG) matrix of Feng et al. (Feng et al., 1984). The values range from 0 (drastic amino acid changes) to 6 (synonymous replacements). **(B)** Probability of occurrence of amino acid replacements calculated according to the PAM-250 substitution matrix.

Next, we studied the frequency and acceptability of the amino acid changes found in the different gene products under study. Firstly, amino acid changes were analyzed using the SG matrix as described previously (Feng et al., 1984) generating values from 0 to 6, with 0 indicating the most drastic amino acid changes and 6 the least, and their relative frequencies shown in Figure 5A. The results show that amino acid changes in Rep were poorly tolerated, since 83.9% of changes have acceptability values above 3, with 38.7% having a value of 6 (synonymous changes). Although the overall degree of amino acid change was similar in all regions, the higher relative frequency of more pronounced changes (with values of 2 or less) found in the CP might indicate that this protein is more tolerant to drastic amino acid changes. We analyzed the probability of amino acid substitution occurrence using a PAM-250 substitution matrix (Feng and Doolittle, 1996). The results shown in Figure 5B indicate that the highest frequency of probable amino acid replacements occurred in Rep and that unlikely amino acid changes were most prevalent in the CP protein. Altogether the results indicate that the Rep protein tends to be more conserved and that the CP protein is more amenable to change.

### Host influences the sequence space explored by begomoviruses

**Figure 6:**
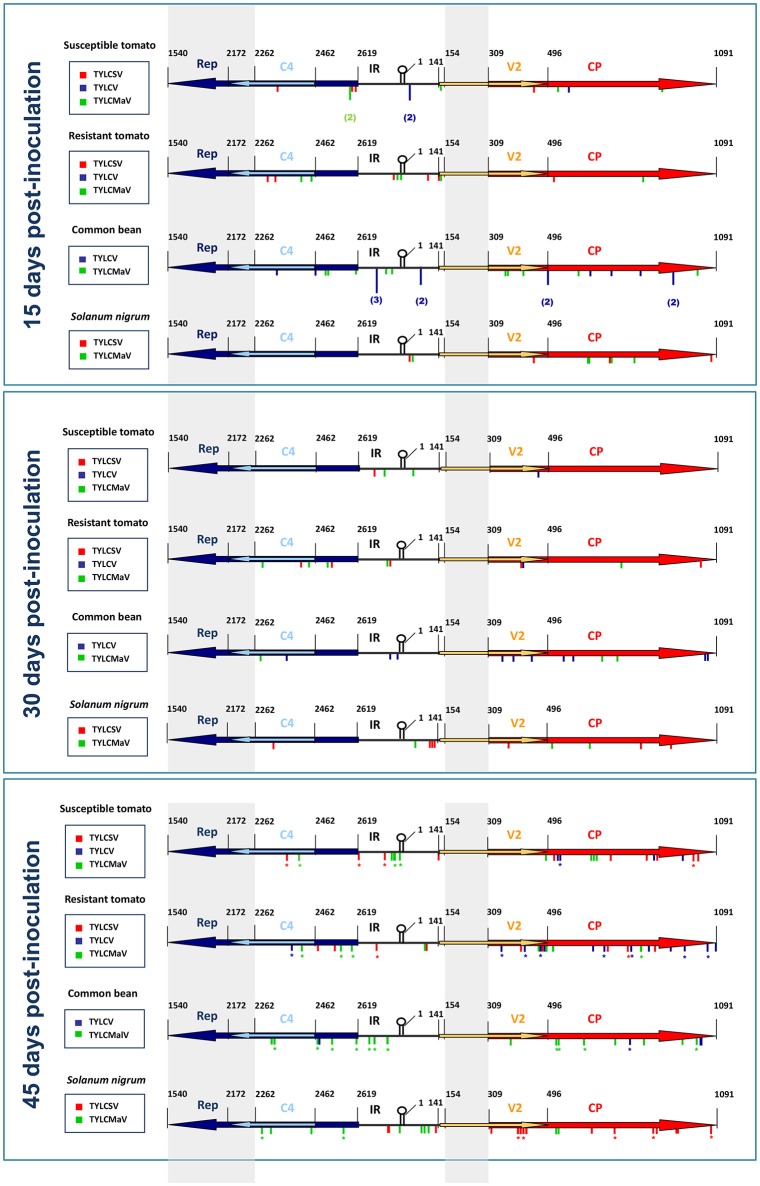
Schematic representation of the distribution of mutations found in quasispecies of time-course infections of tomato yellow leaf curl Sardinia virus (TYLCSV), tomato yellow leaf curl virus (TYLCV) and tomato yellow leaf curl Malaga virus (TYLCMaV) in quasi-isogenic susceptible (ST, *ty-1*/*ty-1*) and resistant (RT, *Ty-1*/*ty-1*) tomato lines, common bean (CB) and *Solanum nigrum* (SN) at 15, 30, and 45 days after inoculation of single sequence variants. Sequenced regions of quasispecies molecular clones include 357 nts of the 5′-end of *Rep*, 200 nts of the 5′-end of *C4* ORF, the IR, 187 nts of the 5′-end of the *V2* ORF and the complete *CP* ORF. Nucleotide positions refer to the TYLCSV sequence [ES:Mur1:92] (Z25751) (Noris et al., 1994). Consensus sequences of the quasispecies were determined for whole viral genomic sequences. Mutations detected in separate quasispecies in the same host/time combination are indicated by the number of times they appeared. Mutations found in replicate samples are denoted with an asterisk.

Sequence variations explored during begomovirus infections seemed to be affected by specific virus-host interactions. Sequence space distribution of the mutations detected in our time-course assays is summarized in Figure 6, which shows the location of the mutations found in begomovirus quasispecies by assay time point, host and begomovirus (TYLCSV, TYLCV, or TYLCMaV). Considering the genomic regions explored at each sampling time, we observed that the tomato *Ty-1* resistance allele did not significantly influence mutation frequency for any ORF, since no significant differences between susceptible tomato and resistant tomato were found at any time point (generalized linear model assuming binary distribution and using logit link function, *P* ≥ 0.05). However, differences between hosts were observed with respect to where mutations occurred over time. Thus, for example, mutation frequency of the *CP* increased for TYLCSV in susceptible tomato between 15 and 45 dpi (*P* = 0.048) and between 30 and 45 dpi (*P* = 0.003), and for TYLCV in resistant tomato between 15 and 45 dpi (*P* = 0.001). This increase in *CP* mutation frequency might be related to the decrease in fitness in tomato. In *S. nigrum*, TYLCMaV (replicates 1 and 2 at 45 dpi) showed a significant increase in the number of mutations in *Rep* between 15 and 45 dpi (*P* = 0.044) and between 30 and 45 dpi (*P* = 0.005) but not in the overlapping *C4* ORF. A random distribution of mutations was found both in *Rep* and *C4* (runs test, *P* > 0.05) so mutational hotspots were ruled out. This result suggests an active exploration of sequence space in *Rep* but not in *C4* for the recombinant virus. No parallel mutations were found in replicate samples at 45 dpi. Our results suggest that begomoviruses explore different sequence space depending on host species, although further studies are needed to confirm this including additional replicates (at 15 and 30 dpi). They also suggest that the presence of the *Ty-1* gene in tomato does not affect how begomoviruses move in sequence space at each time point. Furthermore, it is interesting to note that begomovirus consensus sequences were completely invariant throughout the infection time-courses regardless of mutant spectra or host, and despite the large number of mutations found.

### Direction of selective forces depends on host and is not influenced by *Ty-1*

To assess begomovirus mutant spectra adaptation to hosts, we studied the direction of selective forces on their coding regions. To this end the average rate of synonymous substitutions per synonymous site (d_S_) and of nonsynonymous substitutions per nonsynonymous site (d_NS_) for each mutant spectrum were estimated as reported previously (Pamilo and Bianchi, 1993). Where possible, the d_NS_/d_S_ ratio was also calculated. The results in Table 6 show that d_NS_/d_S_ ratios of begomovirus quasispecies evolving in tomato ranged from 0.149 to 0.616 in *Rep, V2*, and *CP*. Ratios calculated for TYLCSV and TYLCMaV were below 1, suggesting that all three regions were under negative selection, both in susceptible and resistant tomato. However, d_NS_/d_S_ ratios ranged from 0.357 to 1.842 in common bean and from 0.464 to 1.778 in *S. nigrum*, showing that, in these hosts, selective forces acted differentially depending on the coding region. In fact, we observed positive selection (d_NS_/d_S_ > 1) in the *CP* region of both common bean, at 15 and 45 dpi for TYLCV and TYLCMaV infections, respectively, and *S. nigrum*, at 45 and 15 dpi for TYLCSV and TYLCMaV infections, respectively. The results on d_NS_/d_S_ ratios of replicates 1 and 2 at 45 dpi of each virus/host combination were similar, although additional replicates would add more robustness to our conclusions. Altogether, these results suggest differing selective constraints of begomovirus quasispecies in different hosts. Also, it seems that *Ty-1* resistance does not alter the way begomoviruses are selected in tomato, as purifying selection dominates in all the coding regions analyzed. Our data also support positive selection of virus variants in the *CP* region in common bean and *S. nigrum*, whereas *Rep, C4*, and *V2* coding regions are under purifying selection. Positive selection of *CP* region variants in common bean and *S. nigrum* together with higher virus fitness and diversity, and differential mutation distribution occur without alterations to begomovirus consensus sequences. These results suggest that the mutant spectrum plays an important role in begomovirus infections in each host.

**Table 6:** Analysis of diversity at nonsynonymous and synonymous positions in four coding regions of begomovirus time-course infections.

**Host^a^**	**Virus^b^**	**dpi**	**Coding region**											
			***Rep***			***C4***			***V2***			***CP***		
			**d_NS_^c^**	**d_S_^c^**	**d_NS_/d_S_**	**d_NS_**	**d_S_**	**d_NS_/d_S_**	**d_NS_**	**d_S_**	**d_NS_/d_S_**	**d_NS_**	**d_S_**	**d_NS_/d_S_**
ST	TYLCSV	15	0.00037	0.00104	0.356	0.00092	0.00000	-	0.00072	0.00000	-	0.00017	0.00000	-
		30	0.00000	0.00000	0	0.00000	0.00000	0	0.00000	0.00000	0	0.00000	0.00000	0
		45 (1)	0.00000	0.00000	0	0.00000	0.00000	0	0.00000	0.00000	0	0.00053	0.00086	0.616
		45 (2)	0.00000	0.00290	0	0.00080	0.00000	-	0.00000	0.00000	0	0.00052	0.00000	-
	TYLCV	15	0.00000	0.00000	0	0.00000	0.00000	0	0.00000	0.00000	0	0.00022	0.00000	-
		30	0.00000	0.00000	0	0.00000	0.00000	0	0.00075	0.00000	-	0.00015	0.00000	-
		45 (1)	0.00000	0.00000	0	0.00000	0.00000	0	0.00000	0.00000	0	0.00047	0.00000	-
		45 (2)	0.00000	0.00000	0	0.00000	0.00000	-	0.00000	0.00000	0	0.00051	0.00000	-
	TYLCMaV	15	0.00085	0.00000	-	0.00000	0.00000	0	0.00000	0.00000	0	0.00035	0.00000	-
		30	0.00000	0.00000	0	0.00000	0.00000	0	0.00000	0.00000	0	0.00000	0.00000	0
		45 (1)	0.00000	0.00000	0	0.00000	0.00000	0	0.00000	0.00000	0	0.00018	0.00121	0.149
		45 (2)	0.00000	0.00049	0	0.00085	0.00000	-	0.00000	0.00000	0	0.00000	0.00000	0
RT	TYLCSV	15	0.00045	0.00241	0.187	0.00149	0.00000	-	0.00000	0.00000	0	0.00000	0.00037	0
		30	0.00067	0.00000	-	0.00000	0.00000	0	0.00085	0.00000	-	0.00020	0.00037	0.541
		45 (1)	0.00000	0.00243	0	0.00000	0.00000	0	0.00062	0.00351	0.177	0.00062	0.00000	-
		45 (2)	0.00046	0.00000	-	0.00000	0.00000	0	0.00000	0.00000	0	0.00000	0.00000	0
	TYLCV	15	0.00000	0.00000	0	0.00000	0.00000	0	0.00000	0.00000	0	0.00000	0.00000	0
		30	0.00000	0.00000	0	0.00000	0.00000	0	0.00000	0.00174	0	0.00020	0.00000	-
		45 (1)	0.00000	0.00000	0	0.00000	0.00000	0	0.00068	0.00000	-	0.00057	0.00000	-
		45 (2)	0.00000	0.00000	-	0.00133	0.00000	-	0.00171	0.00000	-	0.00211	0.00000	-
	TYLCMaV	15	0.00025	0.00099	0.253	0.00067	0.00000	-	0.00000	0.00000	0	0.00016	0.00000	-
		30	0.00098	0.00000	-	0.00000	0.00168	0	0.00000	0.00000	0	0.00014	0.00000	-
		45 (1)	0.00000	0.00000	0	0.00000	0.00000	0	0.00078	0.00000	-	0.00030	0.00000	-
		45 (2)	0.00039	0.00000	-	0.00067	0.00000	-	0.00000	0.00000	0	0.00000	0.00045	0
CB	TYLCV	15	0.00078	0.00000	-	0.00064	0.00000	-	0.00000	0.00000	0	0.00070	0.00038	1.842
		30	0.00072	0.00000	-	0.00065	0.00182	0.357	0.00073	0.00000	-	0.00076	0.00075	1.013
		45 (1)	0.00000	0.00238	0	0.00000	0.00000	0	0.00000	0.00000	0	0.00015	0.00038	0.395
		45 (2)	0.00000	0.00000	0	0.00000	0.00000	0	0.00000	0.00000	0	0.00031	0.00000	-
	TYLCMaV	15	0.00067	0.00105	0.638	0.00000	0.00000	0	0.00150	0.00158	0.949	0.00091	0.00000	-
		30	0.00045	0.00000	-	0.00000	0.00168	0	0.00000	0.00000	0	0.00030	0.00037	0.811
		45 (1)	0.00000	0.00084	0	0.00081	0.00000	-	0.00000	0.00158	0	0.00055	0.00037	1.486
		45 (2)	0.03800	0.00000	-	0.00356	0.00000	-	0.00000	0.00000	0	0.00078	0.00000	-
SN	TYLCSV	15	0.00000	0.00000	0	0.00000	0.00000	0	0.00068	0.00000	-	0.00040	0.00000	-
		30	0.00047	0.00000	-	0.00000	0.00168	0	0.00095	0.00000	-	0.00056	0.00000	-
		45 (1)	0.00000	0.00000	0	0.00000	0.00000	0	0.00180	0.00388	0.464	0.00095	0.00078	1.218
		45 (2)	0.00000	0.00000	0	0.00000	0.00000	0	0.00186	0.00000	-	0.00036	0.00064	0.563
	TYLCMaV	15	0.00000	0.00000	0	0.00000	0.00000	0	0.00000	0.00000	0	0.00051	0.00035	1.457
		30	0.00000	0.00000	0	0.00000	0.00000	0	0.00000	0.00000	0	0.00000	0.00133	0
		45 (1)	0.00000	0.00121	0	0.00000	0.00000	0	0.00000	0.00000	0	0.00000	0.00000	0
		45 (2)	0.00126	0.00000	-	0.00356	0.00000	-	0.00000	0.00000	0	0.00078	0.00000	-

a *ST, susceptible tomato; RT, Resistant tomato; CB, common bean (Phaseolus vulgaris) and SN, Solanum nigrum*.

b *TYLCSV, tomato yellow leaf curl Sardinia virus; TYLCV, tomato yellow leaf curl virus and TYLCMaV, tomato yellow leaf curl Malaga virus*.

c *d_S_, pairwise synonymous substitutions per synonymous site and d_NS_, nonsynonymous substitutions per nonsynonymous site were calculated according to the Pamilo-Bianchi-Li method based on Kimura's two-parameter model*.

## Discussion

Single-stranded DNA viruses belonging to *Parvoviridae, Geminiviridae*, and *Nanoviridae* have been shown to be as variable as RNA viruses. Moreover, ssDNA virus populations, composed of mutant spectra, have shown strong adaptive capacities similar to those observed in RNA virus populations (Isnard et al., 1998; Nishizawa et al., 1999; López-Bueno et al., 2006; Urbino et al., 2008; Grigoras et al., 2010). The origin of this variability, however, remains unknown for these viruses. Quasispecies-like evolution combined with invariance of the *Rep, C4*, and IR genomic consensus sequences has been previously described for the begomovirus tomato yellow leaf curl China virus (TYLCCNV) (Ge et al., 2007) in naturally infected tomato, and in tomato and *Nicotiana benthamiana* plants experimentally infected with TYLCCNV clones. Here we have looked at the evolution of the whole genomic consensus sequence and mutant spectra variability of the *Rep, C4*, IR*, V2*, and *CP* ORFs of three TYLCD-associated begomoviruses in species of the family *Solanaceae* family, that is, susceptible tomato, resistant tomato carrying the *Ty-1* allele and the wild reservoir *S. nigrum*, as well as to common bean, a species of the distantly-related family *Fabaceae*.

In this work we noticed differences in the fitness of viral isolates between hosts. We used daily Malthusian growth rates (m) to estimate viral fitness since differences in growth rate reflect fitness to a great extent and also because single sequence variants were used to infect hosts (Lalić et al., 2011). Further, because monopartite begomoviruses are restricted to the phloem and replicate only in companion cells perturbations in copy numbers are reduced. Thus, at the last assay time point (45 dpi) fitness in tomato of either TYLCSV or TYLCV was lower than in *S. nigrum* or common bean, respectively. This was clearly evident in tomato plants bearing the *Ty-1* resistance allele, where negative growth rate values were obtained for both TYLCSV and TYLCV, in contrast to TYLCMaV. The fitness of this latter virus remained stable over time in resistant tomato and no significant differences were seen between hosts. Indeed, the statistics support that there are no significant differences in TYLCMaV accumulation between susceptible and resistant tomato. It is interesting that in genotypes with the resistance gene the accumulation of TYLCMaV does not decrease much, compared with the other two TYLCD-associated viruses tested. Its better adaptation in resistant genotypes can have important ecological consequences Therefore, our results corroborate that TYLCMAV is a virus with resistance-breaking properties. This was already observed previously (Monci et al., 2002) and quoted subsequently (García-Arenal and McDonald, 2003). How TYLCMaV, is able to overcome the growth restriction conferred by the *Ty-1* gene, despite the genome sequences of the recombinant virus sharing 99% identity with the corresponding parental virus sequences, is an interesting open question. An improved combination of coding and/or nonconding regions may have been selected after a recombination event in TYLCMaV that better overcame *Ty-1* resistance allele limitations. The *Ty-1* allele has been shown to impair the accumulation of begomoviruses involved in TYLCD (García-Andrés et al., 2009), although, similar to the results obtained here, it has been shown to have little effect on TYLCMaV (Monci et al., 2002). *Ty-1* encodes a γ type RNA-directed RNA polymerase (RDRP) (Verlaan et al., 2013), with *Ty-1* resistance involving enhanced transcriptional gene silencing against TYLCV, as revealed by the enrichment of siRNAs directed against *CP* and *REn*, and cytosine methylation of the *CP* promoter region (Butterbach et al., 2014). We did not detect differences in the complexity or heterogeneity of the three begomovirus mutant spectra in the presence or absence of the *Ty-1* allele for any of the genomic regions analyzed in this study. Nevertheless, in resistant tomato, our data show that a high mutation frequency value was observed for the *C4* region of TYLCSV, the most restricted virus (Monci et al., 2002), suggesting exploration of TYLCSV for better adapted variants to overcome restriction imposed by this host. As *C4* has been shown to be a mild suppressor of gene silencing (Luna et al., 2012) and a viral determinant in overcoming the genetic resistance of some wild tomato species (Tomás et al., 2011), the role of this region in permitting viral accumulation in the presence of *Ty-1* merits further study. Since the sequence of the TYLCMaV isolate used in this work only differs in 7 and 15 nucleotide changes to the corresponding sequences of the parental TYLCV and TYLCSV viruses, respectively, it seems reasonable to hypothesize that the key to overcoming *Ty-1* resistance resides in this particular combination of nucleotide changes, with those detected in the *C4* ORF being prime candidates. Alternatively, an optimized combination of genome fragments to overcome *Ty-1* resistance might be achieved in this virus driven by a recombination event then supporting the importance of modular evolution to adapt to a fluctuating environment (Botstein, 1980; Stavrinides and Guttman, 2004; Belabess et al., 2016).

**Figure 7:**
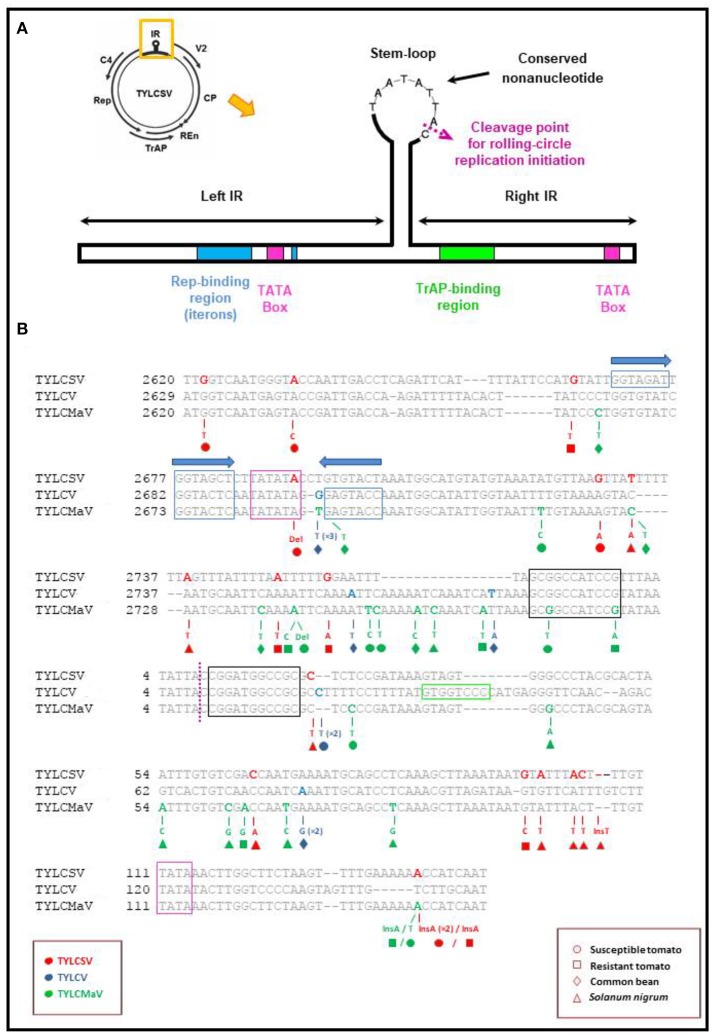
Localization of mutations found in the Intergenic region (IR) of begomovirus quasispecies. **(A)** Schematic representation of the intergenic region of tomato yellow leaf curl-like viruses showing the location of the replication associated (Rep) and the transcription activator (TrAP) protein binding regions, TATA boxes and ERE-like motifs (Bañuelos-Hernández et al., 2012). The nonanucleotide sequence conserved in all geminiviruses and the cleavage site for the initiation of rolling-circle replication in the stem-loop are also shown. **(B)** Representation of the nucleotide mutations found in clones obtained from susceptible (°) and resistant tomato (□), common bean (♢) and *Solanum nigrum* (Δ) plants inoculated with TYLCSV (red), TYLCV (blue) or TYLCMaV (green) infectious clones. Nucleotide numbers on the left-hand side of sequences refer to GenBank accession numbers Z25751 (TYLCSV), AF071228 (TYLCV) and AF271234 (TYLCMaV). Note that Ins141 appeared twice in TYLCSV-infected susceptible tomato at 45 dpi (replicate 1).

The presence of putative low fitness variants in mutant spectra could be the result of compensatory mutations to maintain RNA secondary structure. Further, they may be maintained by complementation or by reduced purifying selection due to a release from the constraint of whitefly transmission in the conducted experiment. Our analysis of mutant spectra identified insertions, deletions, nonsense mutations and readthrough mutations. Such changes often have deleterious or lethal effects on proteins. Others may affect secondary structure or fall in regulatory elements needed to maintain structure for function like some of the mutations found in the IR adjacent to or within a regulatory element (see Table 4 and Figure 7). Among mutations found in TYLCSV, TYLCV and TYLCMaV during infection time-courses in the four hosts (included in Table 4) we highlight the following. The nonsense mutation Q81Stop (C395T) found in the TYLCV V2 protein in infected common bean lacks residues C84 and C86 that have been shown to be essential for PTGS suppression and interaction with the tomato SGS3 protein (Zrachya et al., 2007; Glick et al., 2008). The *Rep* missense mutation C2466T in TYLCMaV-infected resistant tomato, results in an E52K (C2466T) amino acid change between the RCR-1 and RCR-2 DNA binding motifs involved in the specific binding of Rep to DNA (Jupin et al., 1995). The TYLCSV CP V135I and M136I (G711A and G716A, respectively) mutations are in the critical 129–152 region required for effective capsid assembly and transmissibility by *Bemisia tabaci*, the virus vector (Noris et al., 1998; Hallan and Gafni, 2001; Caciagli et al., 2009), although in our experimental design the latter is not a limiting factor. The TYLCMaV CP R18C (C360T) amino acid change is adjacent to R19L, a change which has been shown to deeply impact both TYLCV capsid assembly, as well as CP protein interaction with the tomato nuclear receptor karyopherin α1 and the GroEL protein of *B. tabaci* bacterial endosymbiont (Yaakov et al., 2011). CP mutations Q76E, Y78H, K80T, and R82C (C540G, T546C, A568C, and C585T, respectively) of TYLCV, and Q79H, R80L and D82V (G545T, G547T, and A553T) of TYLCMaV would affect the putative zinc finger motif, thus potentially impairing the ssDNA binding capacity of this protein.

It could be argued that the probability of sequencing genomes with low frequency is expected to be extremely low because of a potential RCA bias (only 0.05% available genomes amplified with RCA) (Gallet et al., 2017) and because of the limited number of sequenced genomes (about 20). In this sense, as a measure to reduce the amplification bias, 3–7 × 10^8^ viral genomes were used as input for RCA, which means that roughly 1.5–3.5 × 10^5^ molecules may have served as template for amplification so no bottlenecks were created at this step. Notwithstanding, there is a previous report with the same experimental nanovirus system (Grigoras et al., 2009) in which the relative quantity of the eight segments of FBNSV, measured by qPCR in total DNA of infected plant tissue, was similar. In addition, the same relative abundance was found in qPCR analysis after performing RCA of the same total DNA or after restriction of the same RCA product with a single-cut enzyme, which ruled out a bias in the RCA. Finally, the analysis of 20 clones per quasispecies may appear insufficient, but assuming each clone is present with a relative frequency of at least 5% within the quasispecies, it means that we might be detecting at least a portion of the most frequent viral variants that are high fitness variants.

Here we have shown that, after the inoculation of a single sequence variant, complex populations arise with a high number of different begomovirus genomes accumulating within each individual plant. We estimate that an average of 2 × 10^11^ begomovirus genomic variants coexist per gram of fresh tissue in apical tomato leaves. Tenfold higher-numbers are reached in common bean and the wild reservoir, *S. nigrum*. This allows begomovirus quasispecies to explore intensively and efficiently sequence space conferring them an enormous capacity to adapt and overcome selective constraints imposed by the host such as the innate or adaptive immune response or post transcriptional gene silencing (Vanitharani et al., 2005; Chen et al., 2010; Raja et al., 2010). The presence of high viral loads of highly diverse geminiviruses is of particular importance for viral emergence. In the infection time-courses, TYLCMaV was able to infect and accumulate sustained high titers within every individual plant species tested, even in the resistant tomato. We showed that quasispecies complexity is especially high in common bean and *S. nigrum*. Common bean is usually employed as a transition host between tomato crops in field rotations, for example in Spain (Sánchez-Campos et al., 1999), which might favor diversification of the begomovirus population between tomato crops. *S. nigrum* is a common wild reservoir in growing areas (García-Andrés et al., 2006), therefore its presence can ensure the maintenance of begomovirus diversity for new epidemics. Importantly, mutations arising in wild reservoirs could be transmitted to crop plants by the insect vector *B. tabaci*, potentially resulting in the emergence and spread of new better fit variants. TYLCMaV can efficiently infect the *Ty-1*-resistant tomato frequently used to contain TYLCD epidemics, as well as the wild host *S. nigrum* and common bean (Noris et al., 1994). Therefore, the high viral diversity of TYLCMaV infections might favor the appearance of ecologically-better adapted emergent variants.

Positive selection in the *CP* region was observed in all three begomovirus quasispecies evolving in common bean and *S. nigrum*, but not in tomato. This finding is unexpected because capsid proteins are involved in vector recognition and particle formation (Noris et al., 1998), and are therefore usually more resistant to selective pressures, as evidenced by lower d_NS_/d_S_ ratios in other viruses (Chare and Holmes, 2004). However, our results indicate that under the conditions studied begomovirus *CP* region incorporated nonsynonymous mutations and accepted more drastic amino acid changes than other genomic regions. Purifying selection might occur in nature during the transmission process, which was not included in our experimental design, as *CP* strongly determines insect recognition (Noris et al., 1998). Nevertheless, under the same experimental conditions, the absence of positive selection in tomato suggests that additional selective constraints on quasispecies development exist in tomato that are absent from the other hosts. Another reason why positive selection in the *CP* region is surprising is because we showed that quasispecies consensus sequences did not change in any of the hosts. It has been suggested that rates of mutation fixation can be higher when viruses are under positive selection, such as when they are adapting to a new host or to cell culture, thus allowing beneficial mutations to become dominant in a short period of time (Carrigan and Knox, 1990; Fares et al., 2001; Acosta-Leal et al., 2011; Domingo et al., 2012). It is worth mentioning that the host change between the plant from which the viral clones were isolated, where they were probably better adapted, and the test plants could constitute such an environmental change that would lead to the evolution and novel exploration of the sequence space. However, in the course of this experiment, we have not found evidence of begomovirus adaptation to different hosts by changing the consensus sequence. Successive passaging of the viruses through the hosts for a longer period of time would have allowed fixation of novel mutations and might have revealed adaptive evolution and perhaps changes in the consensus sequence. Nevertheless, our work suggests that differences in fitness across hosts could be influenced by the distribution of mutations in the mutant spectra. We have shown in previous studies the remarkable continuity of the TYLCSV consensus sequence over an 8-year period (Sánchez-Campos et al., 2002). In this work we have shown that high genetic diversity is generated in all four hosts, especially in common bean and the wild reservoir *S. nigrum* after infection with single sequence variants of three TYLCD-associated begomoviruses in spite of the invariance of the viral consensus sequences. Therefore, our results imply that quasispecies diversity need to be addressed to understand the adaptive potential of these economically important emergent viruses in their different hosts in order to design more durable control strategies.

## Data availability

The raw data supporting the conclusions of this manuscript will be made available by the authors, without undue reservation, to any qualified researcher.

## Author contributions

SS-C, DT, EM, JN-C, and AG-P: conceived and designed the experiments; SS-C, GD-H, DT, and LD-M: performed the experiments; SS-C, GD-H, LD-M, and AG-P: analyzed the data; SS-C, GD-H, LD-M, and AG-P: wrote the main manuscript text and prepared figures. All authors reviewed the manuscript.

### Conflict of interest statement

The authors declare that the research was conducted in the absence of any commercial or financial relationships that could be construed as a potential conflict of interest.
